# Molecular and Aggregate Structural, Thermal, Mechanical and Photophysical Properties of Long-Chain Amide Gelators Containing an α-Diketo Group in the Presence or Absence of a Tertiary Amine Group

**DOI:** 10.3390/gels9010036

**Published:** 2022-12-31

**Authors:** Girishma Grover, Andrea Blake Brothers, Richard G. Weiss

**Affiliations:** 1 Department of Chemistry and Institute for Soft Matter Synthesis and Metrology, Georgetown University, Washington, DC 20057, USA; 2 Department of Chemistry, American University, Washington, DC 20016, USA

**Keywords:** LMOG, gels, α-diketone, tertiary amine, charge transfer, electron transfer, viscoelasticity, photophysics

## Abstract

Three structurally related gelators, each containing octadecyl chains, an α-diketo group at the 9,10 positions, and each with a different N-amide group—isobutyl (DIBA), isopentyl (DIPA) or N-(2-(dimethylamino)ethyl) (DMEA)—have been synthesized. Their neat structures as well as the thermal mechanical, and photophysical properties in their gel states with various liquids have been investigated. The gelator networks of DIBA and DIPA in octane, hexylbenzene and silicone oil consist of bundles of fibers. These gels are partially thixotropic and mechanically, thermally (to above their melting or silicone oil gelation temperatures), and photophysically stable. They are mechanically and thermally stronger than the gels formed with DMEA, the gelator with a tertiary amine group. The lone pair of electrons of the tertiary amine group leads to an intra-molecular or inter-molecular charge-transfer interaction, depending on whether the sample is a solution, sol, or gel. Neat, solid DMEA does not undergo the charge-transfer process because its amino and diketo groups are separated spatially by a large distance in the crystalline state and cannot diffuse into proximity. However, the solution of DIPA upon the addition of triethylamine becomes unstable over time at room temperature in the dark or (more rapidly) when irradiated, which initiates the aforementioned charge-transfer processes. The eventual reaction of the gelators in the presence of a tertiary amine group is ascribed to electron transfer from the lone-pair on nitrogen to an α-diketo group, followed by proton transfer to an oxygen atom on the anion radical of the α-diketo group from a methyl or methylene group attached to the nitrogen atom of the cation radical. Finally, the formation of an α-diketyl radical leads to irreversible electronic and structural changes that are observed over time.

## 1. Introduction

Gelators are molecules that can self-assemble into 3-dimensional (3-D) networks in the presence of a liquid through covalent or non-covalent processes and forces, such as hydrogen-bonding, van der Waals interactions, dipole–dipole interactions, or London dispersion interactions [1]. The liquids are held inside these 3-D networks usually by surface tension and capillary forces [2,3], which prevent their flow in the absence of external stimuli such as thermal, mechanical, photochemical, pH, ultrasonic, etc perturbations [4]. The nature of the gelator-liquid interactions determines whether a stable gel network is formed [5]. Interactions that are too strong or too weak lead to the formation of solutions, weak gels, or precipitates. A sub-class of molecular gels employs low molecular-mass organic gelators (LMOGs), molecules with molecular masses typically less than 3000 Da and capable of gelating solvents at less than ~5 wt% concentrations of the gelator [6]. LMOG-based gels have been studied extensively for decades in order to understand their bulk structure-property relationships [7] and how they may be used, for example, in the remediation of oil-spills [8,9], for toxic dye removal [10], and as vehicles for delivery of cosmetics [11] and drugs, and to facilitate tissue engineering [12].

Aggregation-induced emission enhancement (AIEE) in LMOGs containing a luminogen within their molecular structure is another area of growing interest [13]. Such systems can function, for example, as intermediates in the synthesis of fluorescent films [14], and to construct organic light-emitting devices, chemosensors and biosensors [15]. Zhang et al. recently reported aggregation-induced blue-shifts in the emission of 9,10-dioxooctadecanoic acid (DODA) that depends on the orientation of the α-diketo groups [16]. Furthermore, the presence of an α-diketo group (at the 9 and 10 positions) tends to form an additional dipole–dipole interaction, apart from dimer formation through hydrogen bonding of the carboxylic acid group at the terminal position, thus enhancing the possibility of forming a 3-D network and formation of gels in a variety of polar, as well as low polarity, liquids. The resultant gels also can exhibit enhanced mechanical strength, thixotropic characteristics, and thermoreversibility.

Related to these observations, bimolecular photoinitiator systems comprised of an α-diketone and a tertiary amine have been used as dental restorative materials [17,18]. A mechanism involving a triplet exciplex has been suggested to be the reactive intermediate preceding an electron transfer [17,18], although no specific evidence for such a complex has been shown. Regardless, electron transfer from the tertiary nitrogen lone-pair to the α-dicarbonyl group is the most plausible mechanism for the initial step. The amino alkyl radical formed in a subsequent step acts as the active species for the polymerization reactions. To our knowledge, this electron transfer process has not been explored in an aggregated state or in molecules containing both α-diketo and tertiary amine groups.

**Figure 1:**
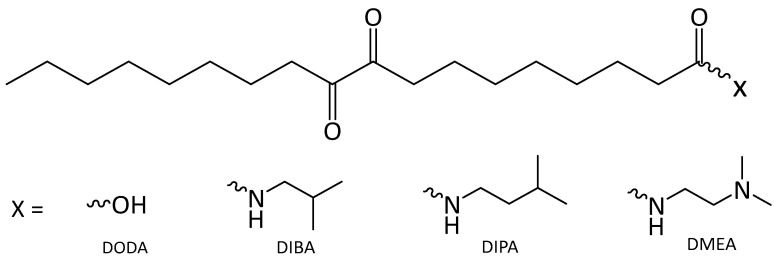
Structures of the alkyl amide derivatives of 9,10-dioxooctadecanoic acid (DODA) examined: N-isobutyl-9,10-dioxooctadecanamide (DIBA), isopentyl-9,10-dioxooctadecanamide (DIPA) and N-(2-(dimethylamino)ethyl)-9,10-dioxooctadecanamide (DMEA).

In this work, we report the aggregation, viscoelastic, and photophysical properties of three alkyl amide derivatives of DODA: N-isobutyl-9,10-dioxooctadecanamide (DIBA), N-isopentyl-9,10-dioxooctadecanamide (DIPA), N-(2-(dimethylamino)ethyl)-9,10-dioxooctadecanamide (DMEA) (Figure 1). All contain an α-diketo group at the 9 and 10 positions, and a secondary alkyl amide. Additionally, DMEA has a tertiary amine group with a pair of electrons that can interact with the α-diketo moiety in several ways. The absence of a terminal carboxylic group in these three gelators and, in its place, the presence of a secondary amide group affects the overall gelation properties of the molecules. Moreover, the presence of a branched alkyl chain, with two different chain lengths and conformations in DIBA and DIPA, can impart additional London dispersion interactions within the aggregates. As both DIPA and DMEA vary only in their tertiary carbon or nitrogen atoms in the terminal alkyl chain, the differences in their gelation properties, such as mechanical and thermal strength as well as packing arrangements in the bulk, provide insights into the effect of the substituent on intramolecular ad intermolecular interactions. 

**Scheme 1:**
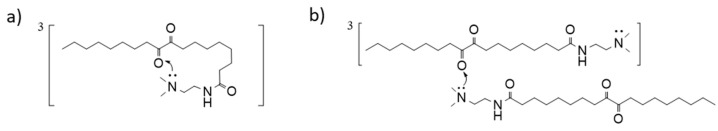
Possible intra-molecular (**a**) and inter-molecular (**b**) orientations for interactions between the excited triplet state of an α-diketo group and the lone-pair of electrons in a tertiary amino group in DMEA leading to electron transfer.

Of special interest here is the nature of the interactions between the tertiary amine and α-diketo groups in DMEA (Scheme 1). If the terminal tertiary amine group is in the immediate proximity of the α-diketo group, either intramolecularly or intermolecularly (i.e., the latter in aggregates), its lone-pair of electrons can interfere with α-diketo electronic transitions, most notably the n-π* ones, thus changing the photophysical properties of the gelator and its resultant gels.

The research presented here focuses on three aspects of these topics: (1) synthesizing, characterizing and applying new classes of gelator molecules with different n-alkyl, tertiary carbon and tertiary amine groups at the terminus of the amide chains to study their gelating abilities in organic solvents; (2) gaining a deeper insight into the thermal, mechanical, and bulk properties of the organogels formed from these gelators; and (3) understanding the photophysical properties of these gelators both in solution states and in aggregates (i.e., gels and sols), especially contrasting when they do (as in DMEA) and do not (as in DIPA) contain a tertiary amino center. In addition, the effects on gelation of the DIPA homologue, DIBA, are explored. 

## 2. Experimental and Procedures

Detailed descriptions of the syntheses and physical properties of DIBA, DIPA and DMEA are included in the Supplementary file.

### 2.1. Materials

Ethyl oleate (Tex Lab Supply, Lubbock, TX, USA, NF/EP-USP grade), acetic anhydride (Sigma-Aldrich, St. Louis, MO, USA, >99%), potassium permanganate (Fisher Scientific, Waltham, MA, USA, certified), sulfuric acid (Fisher Chemical Waltham, MA, USA, ≥95.3%), sodium bisulfate (Fisher Scientific, Waltham, MA, USA, certified), sodium bicarbonate (Sigma-Aldrich, >99.5%), sodium chloride (Fisher Science Education, Waltham, MA, USA, reagent grade), sodium sulfate (anhydrous, Fisher Scientific, Waltham, MA, USA, certified), methanol (Fisher Scientific, Waltham, MA, USA, ≥99.8%), hexane (Fisher Chemical, Waltham, MA, USA, ≥98.5%), ethyl acetate (Fisher Chemical, Waltham, MA, USA, ≥99.5%), tetrahydrofuran (Sigma-Aldrich, St. Louis, MO, USA, ≥99.9%, inhibitor free), triethylamine (Sigma-Aldrich, St. Louis, MO, USA, >99%), ethyl chloroformate (Sigma-Aldrich, St. Louis, MO, USA, 97%), acetic acid (glacial, Fisher Chemical, Waltham, MA, USA, certified ACS), Isobutylamine (Sigma-Aldrich, >99%), isopentylamine (Sigma-Aldrich, St. Louis, MO, USA, >99%), N,N-dimethyl ethylenediamine (Sigma-Aldrich, St. Louis, MO, USA, 99%), dimethyl sulfoxide (Fisher Chemical, Waltham, MA, USA, >99.9%), silicone oil DC 550 (Applied Science Laboratory and DOWSIL™ 550, DOW), carbon tetrachloride (Sigma-Aldrich, St. Louis, MO, USA, 99.9%), octane (Sigma-Aldrich, >98%), decane (Sigma-Aldrich, St. Louis, MO, USA, >99%), 1-butanol (Sigma-Aldrich, St. Louis, MO, USA, >99.5%), acetonitrile (Fisher Chemical, Waltham, MA, USA, 99.9%), benzene (Fisher Scientific, Waltham, MA, USA, 99.9%), toluene (Sigma-Aldrich, St. Louis, MO, USA, 99.9%), chlorobenzene (Sigma-Aldrich, St. Louis, MO, USA, >99.8%), nitrobenzene (Sigma Aldrich, St. Louis, MO, USA, >99%), 1-hexylbenzene (Aldrich, St. Louis, MO, USA, 97%), acetone (Fisher Chemical, Waltham, MA, USA, Histological grade), chloroform-*d* (Cambridge Isotope Laboratories, Tewskbury, MA, USA, 99.8%), benzophenone (Sigma-Aldrich, St. Louis, MO, USA, 99%), isostearyl alcohol (Nissan Chemical, Tokyo, Japan, >99%, Fine Oxocol 180), anhydrous magnesium sulfate (Fisher Chemical, Waltham, MA, USA, Certified Powder), molecular sieves (EMD, 4A, 8–12 mesh beads), p-xylene (Acros, 99%), sodium lumps (Aldrich, St. Louis, MO, USA, assay 99%), cyclohexane (Fisher Science Education, Waltham, MA, USA, reagent Grade), dimethyl formamide (Alfa-Aesar, Ward Hill, MA, USA, >99.8%), sodium metabisulfite (Alfa-Aesar, Ward Hill, MA, USA, 97%), sodium bisulfite (Alfa Aesar, Ward Hill, MA, USA, 97%), and TEM copper grids with carbon/formvar coating (Electron Microscopy Sciences, Hatfield, PA, USA, FCF400-Cu, 400 mesh). 

All reagents were used as received except tetrahydrofuran, which, before use, was dried over 4 Å molecular sieves, refluxed with sodium metal and benzophenone and distilled, to remove water [19].

### 2.2. Instrumentation

^1^H NMR and ^13^C NMR spectra were obtained on a Varian 400 MHz spectrometer operating at 400 MHz (100 MHz for ^13^C NMR) with tetramethylsilane (TMS) as the internal standard. The IR spectra of neat gelators were obtained on a Perkin–Elmer Spectrum One FT-IR spectrometer using an attenuated total reflection accessory (ATR) in a range of 4000–400 cm^−1^ as the average of 8 scans with a resolution of 2 cm^−1^.

Melting points were obtained at a heating rate of ~1 °C/min on a Leitz 585 SM-LUX-POL microscope equipped with crossed polars, a Leitz 350 heating stage and an Omega HH503 microprocessor thermometer attached to a J-K-T thermocouple. Polarized optical micrographs were obtained on a Leitz 585 SM-LUX-POL microscope and images were captured on a Photometrics CCD camera interfaced to a computer using the flame-sealed flattened capillary tubes (Vitro Dynamics). Elemental analyses of neat gelators were performed using a Perkin–Elmer 2400 CHN elemental analyzer with acetanilide as the calibration standard. Differential scanning calorimetry (DSC) scans were obtained on a Q200 differential scanning calorimeter (TA Instruments) in TZero hermetically sealed aluminum pans with a nitrogen flow rate of 50 mL/min. Thermogravimetric analyses (TGA) for DIBA and DIPA were performed on a TA 2910 differential scanning calorimeter interfaced to a TA Thermal Analyst 3100 controller in open aluminum pans under a slow stream of nitrogen. Samples were equilibrated initially at 30 °C and the heating rate was 5 °C/min. TGA for DMEA was performed on a TA Instrument Q5000IR TGA controller under a slow stream of nitrogen in a 100 μL open platinum pan. Samples were equilibrated initially at 23 °C, and the heating rate was 3 °C/min. 

Transmission electron microscopy (TEM) experiments were performed using JEOL JEM-2100 Plus Electron Microscope with an accelerating voltage of 200 kV. Images were analyzed using TEM center for JEM-2100 Plus Software by JEOL. The width of the fibers was obtained from ImageJ software (Image Processing and Analysis in Java) [20,21]. Powder XRD patterns for DIBA and DIPA gels and neat solvents were collected on a Rigaku RAPID Instrument system with Cu Kα X-rays (λ = 1.54 Å), operating at 40 kV and 30 mA, with the collimator at 0.5 mm and a collection time of 10 h. The patterns of the powder gelators, solidified melts of the gelators, DMEA gels in silicone oil and neat silicone oil were collected on Bruker Apex Duo instrument with an Apex II detector using a Cu microfocus source (λ = 1.54 Å) operating at 45 kV and 0.65 mA. Polyamide tubing with an inner diameter of 0.81 mm and outer diameter of 0.86 mm, purchased from Cole-Palmer, was used to mount the samples. The sample collections for powders used four frames of 90 s each in increments of 12° in 2θ from 0°–48°. The sample collections for the gels and neat solvents used three frames of 300 s each in increments of 12° in 2θ from 0°–40°. The data were analyzed using Materials Data JADE (V6.5.26) software [22]. 

Rheology experiments were performed on an Anton Paar-Physica MCR 302 strain-controlled rheometer attached to a Peltier temperature-controlled base plate using a parallel plate geometry (diameter 25 mm) and a 0.5 mm gap between the plates. Data were analyzed using Rheoplus/32 Service V3.62 Software by Anton Paar. 

UV-vis absorption spectra were obtained at room temperature using a Cary 5000 UV-vis-NIR spectrophotometer from Agilent Technologies in quartz cuvettes 1 × 1 cm for solutions or 2 mm capillaries for gels. Steady-state excitations and emissions spectra were recorded using a SYS 2459 Photon Technology International Fluorimeter, with a 75 W Xenon lamp, a Quantum Northwest Peltier temperature controller, an Omega temperature probe and a PTI 814 photomultiplier detector. Felix 32 software by PTI was used for data analyses.

### 2.3. Procedures

#### 2.3.1. ^1^H NMR

Chloroform-*d* was passed through basic alumina before recording the ^1^H NMR spectrum of DMEA to remove traces of DCl/HCl, which is formed over time and can protonate a tertiary amine and alter proton shifts in the NMR spectrum [23]. 

#### 2.3.2. Preparation and Preliminary (Qualitative) Classification of Samples: Gel Formation/Gel Identification

Gels were prepared by placing weighed amounts of DIBA, DIPA or DMEA and a solvent in a 5 mm (id) borosilicate glass tube that was flame-sealed, heated to the boiling point of the solvent (or to 90 °C) and then placed directly into an ice-water bath (~0–2 °C) for 10–15 min. The samples were then kept at room temperature for 1 h (fast-cooled method). The slow-cooled gels were heated as above and then allowed to return to room temperature over 3–4 h while remaining in the slowly cooling water bath. Samples were considered gels if they did not fall over periods of ~1–2 min when the tubes were inverted and if their appearance did not indicate the formation of macro-crystalline aggregates [24].

#### 2.3.3. Critical Gelator Concentrations (CGCs) and Gel Temporal Stabilities

Samples, prepared by the fast-cooling method, were placed in sealed capillaries, inverted and submerged in a stirred water bath, and heated at a rate of 1–2 °C/min to determine the melting ranges reported in the tables. CGCs were taken as the lowest concentrations at which a sample did not fall under the force of gravity at room temperature. Stabilities of fast-cooled gels are the periods of time at room temperature before unperturbed samples fell or phase-separated visibly under the influence of gravity.

#### 2.3.4. Differential Scanning Calorimetry

Aliquots (5–10 mg) of gels from 3 wt% DIBA, DIPA or DMEA in silicone oil, made by the fast-cooling method, were placed in TZero hermetically sealed aluminum pans, equilibrated at 0 °C for 5 min, heated to 110 °C (or 90 °C for DMEA gels), and kept at 110 °C (or 90 °C for DMEA gels) for 5 min. The cooling cycles involved cooling from 110 °C (or 90 °C for DMEA gels) to 0 °C and keeping them at 0 °C for 5 min. The heating and cooling cycles were repeated 3 more times. 

For neat DIBA, DIPA or DMEA, heating cycles started at 40 °C for 1 min, before heating samples to 120 °C (or 95 °C for DMEA) and keeping them at 120 °C (or 95 °C for DMEA) for 10 min. The cooling cycle involved cooling from 120 °C (or 95 °C for DMEA) to 40 °C and keeping the samples at 40 °C for 30 min. The cycles were reproduced 3 more times. Heating and cooling rates were 5 °C/min for all samples.

#### 2.3.5. Transmission Electron Microscopy 

The methodologies for the different aspects of the TEM experiments are described below [25].

Sample preparation method 1: One drop of a sol of 0.05% DIBA in octane was placed on a 400-mesh copper TEM grid supported with carbon/formvar coating and dried in air for 2–3 days (leading to a noticeable solid on the grid). The procedure was repeated 4 more times to deposit more DIBA on the grid. One drop of a sol of 0.5 wt% DIBA in hexylbenzene was placed on a 400-mesh copper TEM grid supported with carbon/formvar coating and dried in air for 2–3 days (Figure S16).

Sample preparation method 2: Small amounts of gel samples of 2 wt% DIBA and DIPA in octane were placed on a glass plate. The same type of TEM grid was brought into contact with the glass plate containing the gel sample momentarily and then the TEM grid was dried in the air for 2–3 days (Figure S17).

#### 2.3.6. Powder X-ray Diffraction Studies 

Gel samples were prepared by placing hot sols of 7 wt% DIBA or DIPA in silicone oil in round, special glass capillaries (W. Müller, Schönwalde, Germany) with 1 mm interior thicknesses. The samples were kept at ~4–6 °C for 15–20 min and kept at room temperature for ~1 h. A gel sample of 7 wt% DMEA in silicone oil was prepared by the fast-cooling method and a polyimide (Kapton) tube with an inner diameter of 0.81 mm was dabbed over the gel to fill the tube with the gel. Powder X-ray data were not collected until/unless the sample was evenly distributed within the tube and without any air bubbles apparent by viewing through a microscope. Samples of neat powders of DIBA, DIPA, and DMEA for powder XRD were prepared by dabbing a Kapton tube over crystals that had been ground into a fine powder. Solidified melts of DIBA, DIPA and DMEA for XRD measurements were prepared by heating them to slightly above their melting temperatures for 10 min and incubating them at room temperature for 1 h before dabbing a Kapton tube over them. 

#### 2.3.7. Rheological Measurements

3 wt% DIBA, DIPA or DMEA gels in silicone oil, prepared by the fast-cooling method, were placed on the lower rheometer plate with a gap of 0.5 mm set between the parallel plates. The linear viscoelastic region (LVR) for strain sweeps was selected as 1 Hz based on examination of the frequency sweeps. Frequency sweep measurements were performed from 0.01 to 100 Hz at a constant strain of 0.01% (i.e., within the LVR; see Section 3.5). Strain sweep measurements were performed from 0.01 to 1000% at a constant frequency of 1 Hz (see Section 3.5). Measurements of thixotropy for a 3 wt% DIBA-silicone oil gel were performed by applying destructive strain (DS: γ = 100% for 10 min or 700% for 13 min, f = 1 Hz), to destroy at least partially the gel network and then observing the recovery in the LVR (γ = 0.1% for 50 min or 0.01% for 33 min; f = 1 Hz). Measurements of thixotropy for a 3 wt% DIBA-silicone oil gel were also performed by applying successive incremental destructive strains (DS: γ = 100%, 200%, 300%, 400%, 500%, 600% and 700% for 3 min, f = 1 Hz), to destroy at least partially the gel network and then observing the recovery in the LVR (γ = 0.1% for 30 min; f = 1 Hz). Measurements of thixotropy for 3 wt% DIPA-silicone oil gel were performed by applying destructive strain (DS: γ = 600% for 13 min, f = 1 Hz), and then observing the recovery in the LVR (γ = 0.01% for 60 min, f = 1 Hz). Measurements of thixotropy for 3 wt% DMEA-silicone oil gel were performed by applying destructive strain (DS: γ = 100% for 10 min, f = 1 Hz) and then observing the recovery in the LVR (γ = 0.01% for 50 min, f = 1 Hz). Experiments following the recovery of G’ and G” were performed sequentially three or four times on the same gel aliquot.

#### 2.3.8. Absorption, Emission, and Excitation Spectra

Photophysical studies on solutions of DIBA, DIPA and DMEA in acetonitrile were conducted at 6 × 10^−3^ M concentrations. A weighed amount of gelator was dissolved in acetonitrile by sonicating for 30 min, heating at 50 °C in a water bath for 1–2 min, then transferring the sample to a quartz cuvette (1 × 1 cm) with a Teflon cap after bubbling nitrogen through the solutions for 5–10 min. All measurements were made within an hour of sample preparation unless mentioned otherwise. For photophysical studies on an oxygen-free solution of 6 × 10^−3^ M DMEA in acetonitrile, the sample was prepared by the same method mentioned above, transferred to a 5 mm (optical path) pyrex capillary and flame-sealed after three freeze-pump-thaw cycles at 20 mTorr. The measurements were made at room temperature. 

In one experiment, triethylamine (TEA, 7.30 mg, 0.072 mmol) was added to a solution of 6 × 10^−3^ M DIPA in acetonitrile (6.90 mg, 0.018 mmol) that had been kept at room temperature for 14 days, and then the mixture was sonicated for 2 h and used to record excitation and emission spectra. 

Photophysical studies on sols and gels of DIPA and DMEA in silicone oil were conducted at 3 wt% concentrations. Gels were prepared by the fast-cooling method in 2 mm (optical path) pyrex capillaries and sealed with a rubber cap. All excitation and emission spectra were recorded at increments of 1 nm in 2 s intervals. The flattened pyrex capillaries were placed inside a 1 cm × 1 cm quartz cuvette containing silicone oil and the Peltier temperature controller attached to the fluorometer was used to heat and cool the samples. The temperature was calibrated by placing a thermometer inside the cuvette containing the silicone oil. It showed less than ±2 °C differences from the Peltier settings.

#### 2.3.9. Density Functional Theory Calculations

The lengths of conformationally extended single molecules of DIBA, DIPA and DMEA were calculated by the B3LYP/6-31G(d,p) DFT method using the Gaussian 16 package [26,27,28,29,30,31,32]. The van der Waals radii of hydrogen atoms (1.2 Å) at each chain terminus was added to the calculated lengths. 

## 3. Results and Discussion

### 3.1. Gelation Properties 

Experiments performed using DIBA, DIPA and DMEA with several low-polarity solvents resulted in gelation (Table 1 and Table S1). Previously, Zhang et al. reported the gelation capabilities of DODA. Unlike DIBA, DIPA and DMEA, DODA has a carboxylic acid group at a chain terminus. It gelated a variety of high and low-polarity solvents [16]. The absence of an acid group at the terminus of the molecule decreases the capability of DIBA, DIPA and DMEA to interact with polar solvent molecules through hydrogen bonding, and, thus, being incapable of gelating them. Although DIBA, DIPA and DMEA can form gels with the polar aprotic solvent, dimethyl sulfoxide (DMSO), due to dipole–dipole interactions between the α-diketo groups of the gelators and the sulfoxide groups [33], the gels formed are weak due to competing for inter-molecular dipole–dipole interactions within the gelator.

Furthermore, the samples in 6 mm (i.d.) tubes were inverted and tapped twice gently to determine qualitatively the strength of the gels formed using DIBA, DIPA and DMEA. If a gel started to flow upon being tapped, it was considered weak; if it remained unchanged, it was considered a strong gel. DIBA and DIPA formed strong gels with octane, silicone oil and hexylbenzene; of these, a strong gel formed only with silicone oil as the liquid when DMEA was the gelator. DMEA formed weak gels with most of the liquids in Table 1. Thus, the presence of a tertiary amine group in DMEA changes the gelator-solvent interactions, making its gels weaker than those of DIBA or DIPA. 

The stabilities of the DMSO, hexylbenzene, octane and silicone oil gels were observed over time at room temperature (Table 2). The gels with silicone oil persisted when the samples were inverted, and they did not phase-separate for periods up to 6 weeks with DMEA and 8 weeks with DIBA and DIPA. Experiments focused more on gels with silicone oil due to their longer periods of stability and the high boiling point of silicone oil (which avoids its evaporation during the observation periods). Furthermore, the silicone oil gels formed at very low gelator concentrations (i.e., critical gelator concentrations, CGCs, of 0.6 wt%). Phase separation was observed in each case at 0.4 wt%.

### 3.2. Thermal Properties

**Figure 2:**
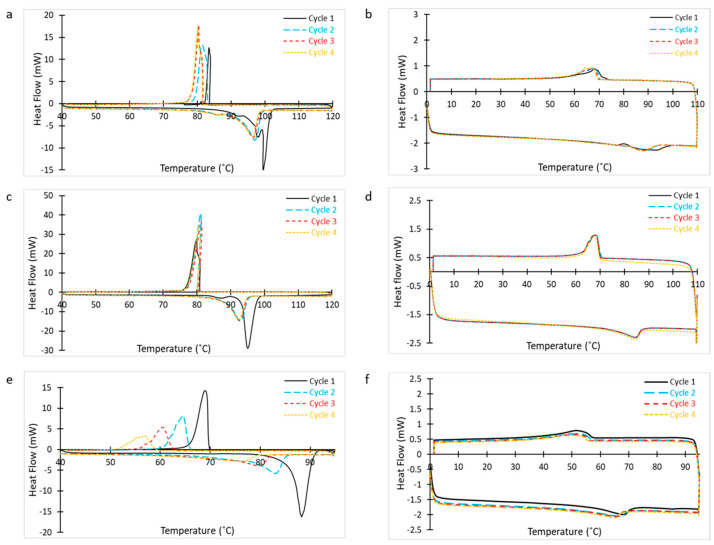
DSC thermograms: (**a**) neat DIBA; (**b**) 3 wt% DIBA-silicone oil gel; (**c**) neat DIPA; (**d**) 3 wt% DIPA-silicone oil gel; (**e**) neat DMEA; (**f**) 3 wt% DMEA-silicone oil gel. The measurements were repeated four times on one sample aliquot. Both heating (negative heat flows) and cooling (positive heat flows) cycles are shown.

Differential scanning calorimetry (DSC) thermograms of neat DIBA and DIPA show one endotherm (melting point) and one exotherm and, thus, the formation of crystalline networks upon cooling (Figure 2a,c). The first and the second heating profiles show slight differences due to the gel preparation method. The gels were prepared by the fast-cooling method for the first heating cycle. However, after converting the gel-to-sol upon heating, the sample was slowly cooled to 5 °C/min in the DSC instrument to reform a gel. Note that gels prepared inside the instrument are slow-cooled. Thus, all successive heating cycles and cooling cycles were reproducible. 

DSC curves for 3 wt% DIBA and DIPA gels in silicone oil (Figure 2b,d) exhibit a single endotherm during heating, indicating that the gels do not undergo association/dissociation before converting to sol phases and, upon cooling, crystallize into the same crystalline gel phase in successive cycles. Since multiple cycles are reproducible, gel-to-sol and sol-to-gel conversions are thermoreversible and take place through the same phases.

Successive heating and cooling cycles of neat DMEA were not reproducible (Figure 2e). According to thermogravimetric analysis, neat DMEA (Figure S14b) is stable to ~100 °C. To obtain thermograms, DMEA was heated to 90° C and equilibrated there for 5 min before cooling; this protocol may have led to slight decomposition. On the other hand, the 3 wt% DMEA-silicone oil gel was reproducible in successive heating cycles (Figure 2f) when equilibrated at 95 °C for 10 min. Due to the low concentration of gelator in the 3 wt% DMEA-silicone oil gel, any decomposition that may have occurred was below our level of detection, and the gel-to-sol transition appeared to be thermoreversible. 

The gel-to-sol transition temperatures and the melting temperatures of the gelators determined by optical microscopy (Table 3 and Table 4) are comparable to the temperatures found by DSC measurements. The transition temperatures for the DIBA and DIPA gels (~93 °C and ~85 °C, respectively) are higher than those for the DMEA gels (~68 °C). Disregarding the structural instability of DMEA, this result indicates that the DIBA and DIPA gels are thermodynamically more stable than the DMEA gel, at least at the 3 wt% concentrations employed for this experiment. This enhanced stability may be due to the formation of N-H---N: inter-molecular hydrogen bonds between the amide and tertiary amine groups in DMEA, which competes with N-H---O=C inter-molecular hydrogen bonds between adjacent amide groups that strengthen gelator-gelator interactions and weakens gelator-solvent interactions. 

### 3.3. Morphologies from Polarized Optical Micrographs and Transmission Electron Microscopy

**Figure 3:**
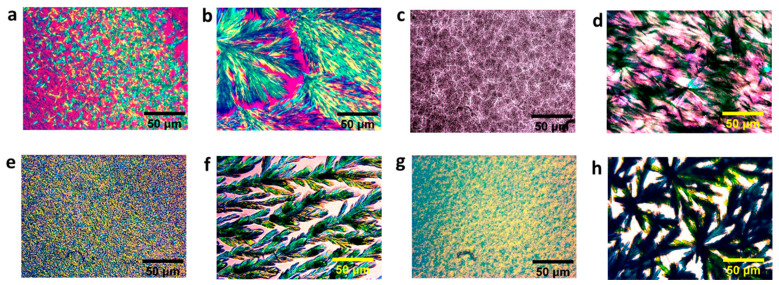
Polarizing optical micrographs at room temperature: 5 wt% DIBA in hexylbenzene gel prepared by (**a**) fast-cooling method; (**b**) slow-cooling method, 5 wt% DIBA in octane gel prepared by (**c**) fast-cooling method; (**d**) slow-cooling method, 5 wt% DMEA in hexylbenzene gel prepared by (**e**) fast-cooling method; (**f**) slow-cooling method; 5 wt% DMEA in octane gel prepared by (**g**) fast-cooling method and (**h**) slow-cooling method. The scale bars represent 50 μm. DIBA and DMEA gels were prepared in flame-sealed flattened capillary tubes of interior thicknesses of 1 mm (octane gels), or 0.5 mm (hexylbenzene gels).

Figure 3 and Figure S15 show polarized optical microscope (POM) images of gels from 5 wt% DIBA, DIPA and DMEA in hexylbenzene, octane and silicone oil. Gels prepared by the fast-cooling method show networks of small fibers due to multiple nucleation sites (Figure 3a,c,e,g and Figure S15a,c,e,g,i). Gels prepared by the slow-cooling method consisted of long fibers. Those formed in octane were spherulitic (Figure 3d,h), and the networks in hexylbenzene showed feather-like fibers (Figure 3b,f).

**Figure 4:**
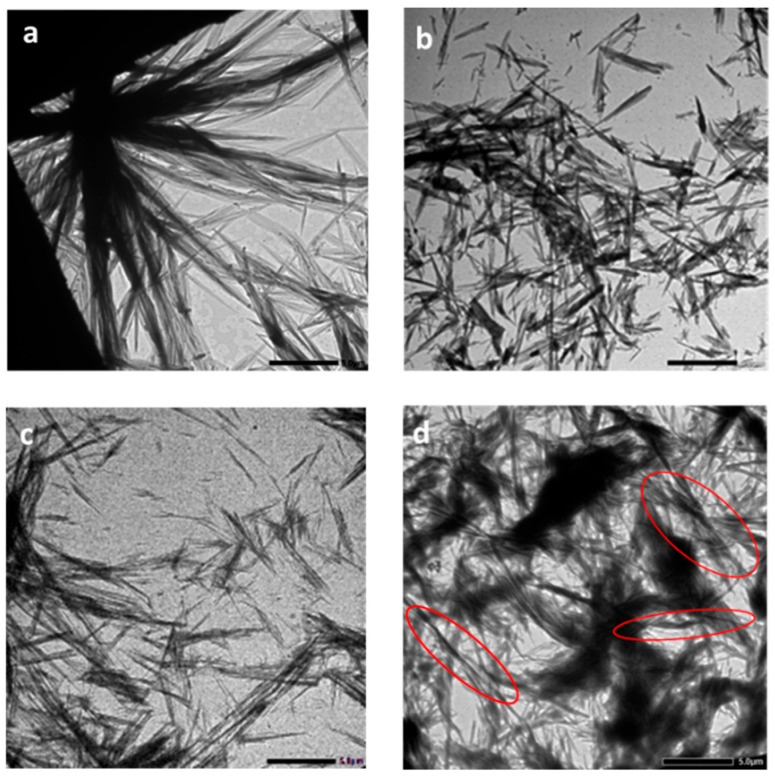
TEM images: (**a**) 0.05 wt% DIBA in octane (solution/sol); (**b**) 2 wt% DIBA in octane (gel); (**c**) 2 wt% DIPA in octane (gel); and (**d**) 0.5 wt% DIBA in hexylbenzene (solution/sol). The scale bar represents 5 μm. The red circles in image (**d**) emphasize regions where twisting is obvious.

The morphologies of these gels on the nanoscale were obtained from transmission electron microscopy (TEM) images (Figure 4). The widths of the fibers in dried sols of 0.05 wt% DIBA in octane and 0.5 wt% DIBA in hexylbenzene and from gels of 2 wt% DIBA and DIPA in octane are shown in Tables S2–S5. The fibers were sorted in 20 nm (or 0.02 μm) width ranges. The number of observations (in the 20 nm range), plotted as a function of fiber widths (μm), are shown in Figures S18–S21 [34]. As interpreted, the plot of the number of observations as a function of fiber widths for 0.5 wt% DIBA in hexylbenzene (Figure S19) shows a range of individual fibers (0.05 μm–0.09 μm), doubled fibers (0.09 μm–0.15 μm), triple fibers (0.15 μm–0.23 μm) and quadruple fibers (0.23 μm–0.31 μm). For plots of 0.05 wt% or 2 wt% DIBA in octane and 2 wt% DIPA in octane, the distinction of individual, double, triple, or quadruple fibers was difficult due to the limited number of observations (Figures S18, S20 and S21). The measured fiber widths for 0.05 wt% or 2 wt% DIBA in octane are comparable (Tables S2 and S4). Both 2 wt% DIBA and 2 wt% DIPA in octane show similar fiber widths (Tables S4 and S5). Thus, the fiber widths do not show a clear dependence on the concentrations of the sols/gels or the number of carbon atoms in the amide part of the gelators. The fiber widths for 0.5 wt% DIBA in hexylbenzene are ~100 nm narrower (Table S3) than those of 0.05 wt% or 2 wt% DIBA in octane and 2 wt% DIPA in octane. Furthermore, twisting of the fibers was observed only in the sol of 0.5 wt% DIBA in hexylbenzene. These results indicate that the fiber widths and degrees of twisting are dependent on the nature of the solvent used for gelation [35]. No twisting of fibers and larger widths were observed with octane as the gelation solvent. However, the origin of the solvent dependence on twisting and fiber widths has not been explored in detail. 

### 3.4. Molecular-Packing Arrangements by PXRD

**Figure 5:**
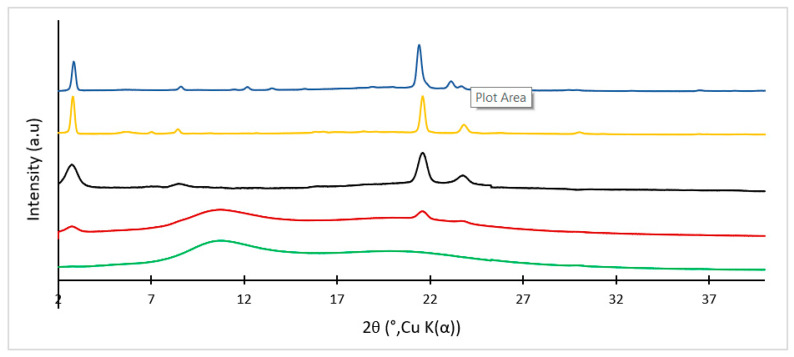
Powder X-ray diffraction patterns: a solidified melt of DIBA (blue), neat unmelted DIBA (yellow), empirically subtracted diffraction patterns of silicone oil from a 7 wt% DIBA in silicone oil (black) and from a 7 wt% DIBA in silicone oil gel (red), and from silicone oil (green). The Y-axes are offset vertically.

The molecular packing arrangements of the gels in silicone oil and their respective gelators were investigated by powder X-ray diffraction (PXRD). Diffraction from neat silicone oil was subtracted empirically from the diffraction patterns of the silicone oil gels with 7 wt% DIBA, DIPA or DMEA (Figure 5, Figures S22 and S23). The 2θ values of the resultant diffraction patterns of the gels are similar to those of their respective neat gelators. However, due to the small number of diffraction peaks for the gels and neat gelators, it was not possible to assign the unit cell types. The *d*-spacings from Bragg’s law (Equation (1)) for neat gelators, gels, and solidified melts of gelators are tabulated in Tables S6–S8 [36]:

nλ = 2*d*sinθ (1)

λ is the wavelength of the x-ray radiation (λ_Cu_ = 1.54 Å), *d* is the spacing of the crystal layers, θ is the incident angle, and n is an integer.

The *d*-spacing (as well as 2θ) values for the solidified melts of gelators and their respective neat gelators are comparable, indicating similar packing arrangements upon melting the gelators and cooling them to room temperature (Figure 5, Figures S22 and S23) [37]. Furthermore, as expected, the diffraction patterns for the 7 wt% DIBA, DIPA and DMEA gels in silicone oil show fewer peaks than their respective neat gelators or their solidified melts due to the lower gelator concentrations and (possibly) greater disorder in the solid networks than in the neat solids. Additionally, the 2θ values and the *d*-spacings for the gels are similar to those of their neat gelators, indicating similar packing arrangements of DIBA, DIPA and DMEA in their neat and gel states.

The lowest angle 2θ peaks (with the maximum *d* spacings) for neat DIBA (31.7 Å), DIPA (33.4 Å) and DMEA (34.2 Å) are near to their extended lengths, which were calculated by DFT to be 28.8 Å, 29.5 Å and 30.9 Å, respectively (Figure S24). For neat DIBA, the *d*-spacings calculated using Bragg’s law (31.7:15.4:10.5:5.5 Å) follow a 1:$\frac{1}{2}$:$\frac{1}{3}$:$\frac{1}{6}$ progression with a deviation of ± 0.5 Å (Table S6). While for neat DIPA, the *d*-spacing of 33.4: 15.7: 11.1: 7.4: 4.9 Å corresponds to a 1:$\frac{1}{2}$:$\frac{1}{3}$:$\frac{1}{4}$:$\frac{1}{6}$ progression with a deviation of ± 1.0 Å (Table S7). Similarly, for neat DMEA, the *d*-spacing values of 34.2: 15.8: 11.1: 9.1: 5.6 Å correspond to a 1:$\frac{1}{2}$:$\frac{1}{3}$:$\frac{1}{4}$:$\frac{1}{6}$ progression with a deviation of ± 1.3 Å (Table S8). On this basis, the packing arrangements of all of the neat gelators appear to be lamellar [37,38]. 

The data indicate no significant differences among the packing arrangements of DIBA, DIPA and DMEA, apart from slight increases attributable to the chain lengths of the amide group portions and preferred small conformational differences (i.e., differences in the alignment of the head groups attached to amide; Figure S24): DIBA and DIPA differ in chain length and possibly the orientations of their amide chains within the aggregates; DIPA and DMEA are different in that the end groups are either a tertiary carbon atom or a tertiary amine nitrogen atom with a lone pair of electrons. 

Additionally, the mean size of the ordered (crystalline) domains (L) was calculated using the Scherrer equation (Equation (2)) [39], where K is a dimensionless shape factor, λ is the X-ray wavelength (1.54 Å), β is the line width at half height (FWHM) in radians, and θ is the Bragg angle. The mean sizes of the ordered crystallites (L) were obtained using JADE software with K values near 1 (±0.05).

(2) $$\beta \;=\;\frac{K\lambda }{L\;\cos\theta }$$

Based on the L values in Table 5, stronger gelator-solvent interactions lead to smaller mean crystallite sizes of the gelator molecules and larger amorphous domains within the gels [40]. The crystallite sizes for gels using different gelators varied and their sizes were in the order DMEA > DIPA > DIBA. 

### 3.5. Viscoelastic Properties of Gels

**Figure 6:**
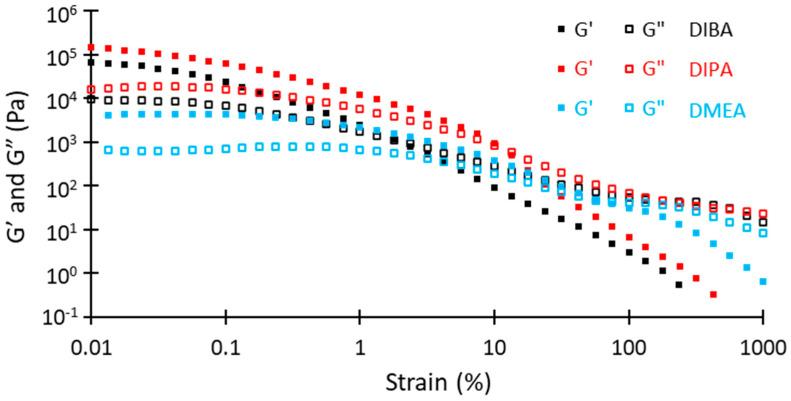
Log–log strain sweeps (1 Hz frequency) at 25 °C: 3 wt% DIBA-silicone oil gel (**black**); 3 wt% DIPA-silicone oil gel (**red**); and 3 wt% DMEA-silicone oil gel (**blue**).

The mechanical properties and stabilities [41] of 3 wt% DIBA, DIPA and DMEA in their silicone oil gels were investigated using rheology. The linear viscoelastic regions (LVR) for the gels were determined by plotting G’ (the storage moduli) and G” (the loss moduli) as a function of strain amplitude with a constant frequency of 1 Hz (Figure 6) [42]. Tan δ (the loss or damping factor), defined as the ratio between G” and G’ (Equation (3)), is a useful parameter to ascertain the viscoelastic behavior of a gel [43].

(3) $$\tan\;\delta \;=\;\frac{G”}{G’}$$

The cross-over points [44] (at tan δ = 1, below which a system behaves like a solid and above which it behaves like a fluid) for the gels, were between 1.8 and 2.4% strain for the DIBA gels (Table S9). The cross-over point for the DIPA gel was between 10.0 and 13.4% and for the DMEA gel, it was between 56.3 and 75.1% (Table S9). Tan δ increased exponentially as the strain increased for DIBA, DIPA and DMEA in silicone oil gels and these gels started to become liquid-like at tan δ >1 (Figure S25). At 3 wt%, the cross-over points of the gels of DIBA, DIPA and DMEA in silicone oil) are in the order DIBA < DIPA < DMEA (Table S9). Thus, the stiffness of the DIBA gel is highest; non-covalent interactions between DIBA and silicone oil became weak on the application of extremely low strains (1.8%), where the gel converted to a sol. As proposed previously, the presence of the tertiary amine group in DMEA induces a conformational change at the amide part of the gelator, thus affecting the gelator-solvent interactions. The gels of DMEA were capable of withstanding higher strains (~56%) than those of DIPA (~10%) before converting to sols.

**Figure 7:**
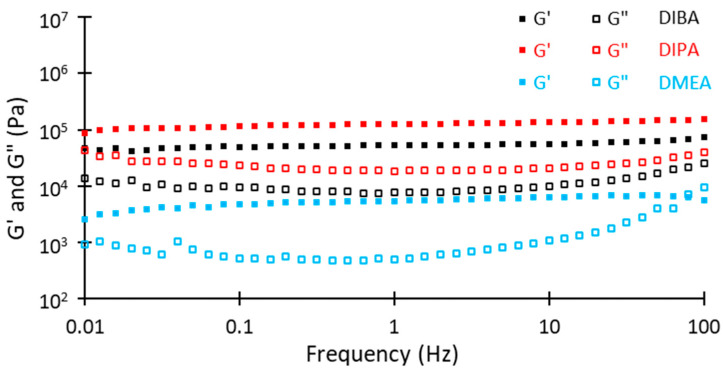
Log–log frequency sweeps (0.01% strain) at 25 °C: 3 wt% DIBA-silicone oil gel (**black**); 3 wt% DIPA-silicone oil gel (**red**) and 3 wt% DMEA-silicone oil gel (**blue**).

The magnitude of G’ for silicone oil gels with 3 wt% DIBA or DIPA was nearly independent of the applied frequency (Figure 7). The tan δ versus frequency plot for these gels (Figure S26) shows a parabolic curve where the DIBA and DIPA gels are solid-like with tan δ < 1 at all frequencies [45]. Furthermore, the high values of G’ (>10^4^ Pa) indicate the presence of strong interactions within these gel networks. The magnitude of G’ in the DIPA gel was slightly higher than that of the DIBA gel, which we tentatively attribute to conformational changes at the amide part caused by the different number of carbon atoms in the alkyl chains attached at the amide nitrogen. On the other hand, the frequency sweep for the corresponding DMEA gel (Figure 7) was dependent on the frequency applied; the DMEA gel was solid-like with tan δ < 1 between 0.1 and 63.1 Hz and fluid-like with tan δ > 1 between 79.4 Hz and 100 Hz (Figure S26). At higher frequencies, the viscoelastic behavior of the DMEA gel was affected and G’ became greater than G” due to the inability of the DMEA gel to rearrange and retain its solid-like behavior at shorter time scales. This indicates that the presence of the tertiary amine group in DMEA weakens the gelator-solvent interactions more than does the terminal tertiary carbon atoms in DIBA and DIPA. 

Some of the gels regained part of their viscoelasticity upon cessation of the application of destructive strain (DS) [46]; they are thixotropic. The 3 wt% DIBA in silicone oil gel (whose solid-like behavior was disrupted upon application of strain greater than 2.4%) was subjected to a 100% DS. After returning to a LVR strain level, the gel recovered repeatedly more than 90% of its initial G’ value (Figure S27 and Table S10). When the same gel was subjected to 700% strain, it regained only 10% of its initial G’ value found in the first cycle and less than 10% in 3 successive cycles (Figure S28 and Table S10). These results indicate that even though the gel began to behave like a liquid at 100% strain, it still retained a considerable amount of gelator–gelator and gelator–solvent interactions in the bulk and was able to reform a strong gel network upon cessation of destructive strain. However, at 700% strain, the associative gelator interactions were almost completely destroyed, making it difficult for the gel/sol to regain its viscoelastic properties. To understand better this behavior, the successive magnitudes of the DS were increased after each recovery cycle from 100% to 700% (Figure S29 and Table S11). In each, more than 70% of the magnitude of the *previous* G’ was recovered. Overall, the recovery in each cycle decreased 10–15% of the initial G’ in consecutive cycles when the applied DSs were 100, 200, 300, 400 and 500% and 5–10% in each cycle when the DSs were 600% and 700%. Thus, the recovery of viscoelasticity (i.e., magnitude of G’) decreased in each successive cycle, destroying the network incrementally and irreversibly.

The 3 wt% DIPA in silicone oil gel at 600% DS recovered less than 2% of its initial G’ value upon cessation of DS during four cycles (Figure S30, Table S10). Thus, even the DIPA gel was destroyed to a significant extent at very high DS. For DMEA gels, on the other hand, 100% DS was sufficient to destroy the gel network to an extent where only ~10% of the initial G’ was recovered in the first cycle (Figure S31 and Table S10). Thus, at 100% DS, the DIBA in silicone oil gel was thixotropic, whereas the DMEA gel was rendered mechanically less stable due to the presence of the tertiary amine group. 

### 3.6. Photophysical Properties of Gelators in Solution/Sol and Gel States

As a neat powder, stored under a nitrogen atmosphere at 4–6 °C, DMEA underwent no discernible changes in its photophysical or structural properties over a period of one year. As mentioned, neat, solid DMEA packs in a lamellar arrangement in which there is a large separation between tertiary amine and α-diketo groups both intermolecularly and intramolecularly. When DMEA is freshly dissolved in acetonitrile (6 × 10^−3^ M), its excitation spectrum showed one peak at 419 nm and a shoulder, which over a period of hours to days, developed another peak at 364 nm (λ_em_ = 479 nm). Thus, the process responsible for the transformation of DMEA must involve the ability of the tertiary amine and α-diketo groups to interact over short distances. 

**Figure 8:**
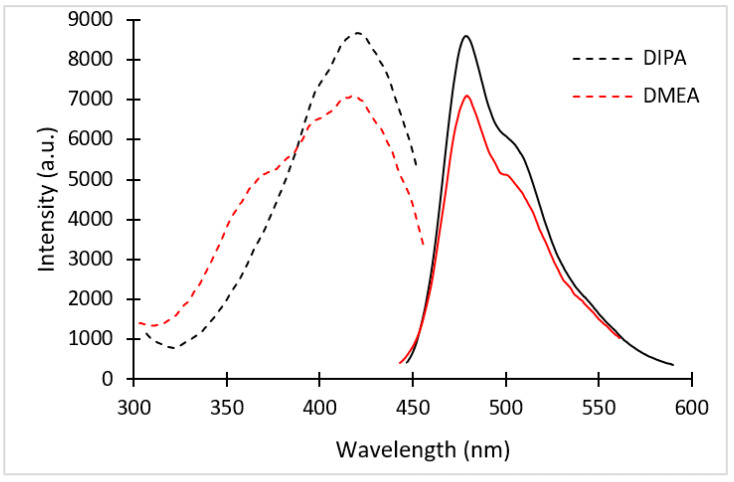
Fluorescence spectra of 6 × 10^−3^ M DIPA in acetonitrile: excitation (λ_em_ = 479 nm; black, dotted line), emission at λ_ex_ = 419 nm (black, solid line) and of 6 × 10^−3^ M DMEA in acetonitrile: excitation (λ_em_ = 479 nm; red, dotted line) and emission at (λ_ex_ = 419 nm; red, solid line) within 1 day after the solution was prepared. All measurements were obtained at room temperature in a 1 × 1 cm (optical path) quartz cuvette.

The absorption spectra of 6 × 10^−3^ M DIBA, DIPA and DMEA solutions in acetonitrile show a broad π-π* transition from 250 to 300 nm, and a broad n-π* transition at 350–490 nm corresponding to absorption bands of an α-diketo group (Figure S32) [47]. Upon excitation at 419 nm, both DIPA and DMEA solutions showed emission spectra with a maximum at 479 nm and a shoulder at ~506 nm (Figure 8). These emission spectra resemble that from DODA in solution [16]. 

The excitation spectra (λ_em_ = 479 nm) of DIPA solution displayed a maximum at 419 nm with a shoulder at ~396 nm. However, as mentioned above, the DMEA solution developed another shoulder at ~364 nm along with peaks at 419 nm and ~396 nm (Figure 8). The shoulder at ~364 nm is absent in both DODA [16] and DIPA solutions (Figure 8). To probe the photophysical changes, excitation, and emission spectra (λ_em_ = 479 nm and λ_ex_ = 419 nm, respectively) were recorded at various times after the preparation of a of 6 × 10^−3^ M DMEA solution in acetonitrile (Figure S33). Two days after preparation, the intensity of the excitation peak at ~364 nm had increased with respect to the peak at 419 nm. By contrast, the excitation and emission spectra of a 6 × 10^−3^ M DIPA solution in acetonitrile were unchanged for as long as 13 days after sample preparation (Figure S34). Even after the removal of oxygen by the freeze-pump-thaw technique, and flame-sealing the DMEA solution and storing it in the dark, the excitation spectrum (λ_em_ = 479 nm) showed the new peak at ~364 nm and a broad peak centered at ~419 nm 4 days after sample preparation (Figure S35). 

Changes in photophysical properties in the DMEA solution over time were suspected to be related to an electron transfer process in the ground state and, as mentioned, changes were noted in the dark as well as upon irradiation (*vide infra*). No photophysical changes were observed in DIPA, which contains an α-diketo group but lacks a tertiary amine group. To determine whether the spectral changes in DMEA are a result of specific interactions between the α-diketo and tertiary amine groups, a 4-molar excess of triethylamine (TEA) was added to a 6 × 10^−3^ M DIPA in acetonitrile solution and the photophysical changes were tracked over time (Figure S36). No major changes in excitation (λ_em_ = 479 nm) and emission spectra (λ_ex_ = 419 nm) were observed during the first 3 days after the addition of TEA when the sample was kept in the dark. However, after 8 days, the excitation spectrum at λ_em_ = 479 nm showed a decrease in the intensity of the excitation peak at 419 nm and the emergence of a new peak centered at ~364 nm. No changes were observed in the emission spectrum at λ_ex_ = 419 nm. These photophysical changes resembled those observed in DMEA after 2 days (Figure S33a). 

One possible source of these photophysical changes is a Norrish Type II reaction in which the hydrogen abstraction can occur from a C-H bond γ to a diketo group of DIBA, DIPA or DMEA in solution. In that regard, Turro and Lee reported the formation of cyclobutanones upon irradiation of pentan-2,3-dione [48]. However, the reaction has a very low quantum efficiency. Under the conditions of our experiments, with very low light intensities, the shapes and intensities of the emissions from DIPA and DIBA are unchanged in solutions over protracted periods. Thus, Norrish Type II reactions appear to be unimportant in the systems under investigation here.

Another possibility is fluorescence and phosphorescence quenching of excited singlets and triplets of the α-diketo group by the tertiary amine group. Turro and Engel reported quenching of biacetyl fluorescence and phosphorescence by several aliphatic tertiary amines in solution [49]. The rates of quenching for both were enhanced by increasing the polarity of the solvent. Okutsu et al., followed the changes in photophysical properties of benzil, another α-diketone, in the presence of TEA after laser flash excitation at 355 nm [50]. In the absence of TEA, benzil developed a transient absorption at 480 nm. Upon the addition of TEA, another peak at 370 nm, corresponding to benzil ketyl radical, appeared and increased in intensity with decreased intensity of the 480 nm peak upon increasing the time of the laser flash. A contact-ion pair between the benzil ketyl anion and TEA cation was observed as a low-intensity, broad peak at 600 nm. By analogy, we attribute the excitation peak at ~364 nm for the DMEA solution to the DMEA ketyl radical. The short-lived DMEA anion radical cannot be observed in the excitation spectrum due to instrument limitations; excitation spectra were measured up to 450 nm at λ_em_ = 479 nm. 

**Scheme 2:**
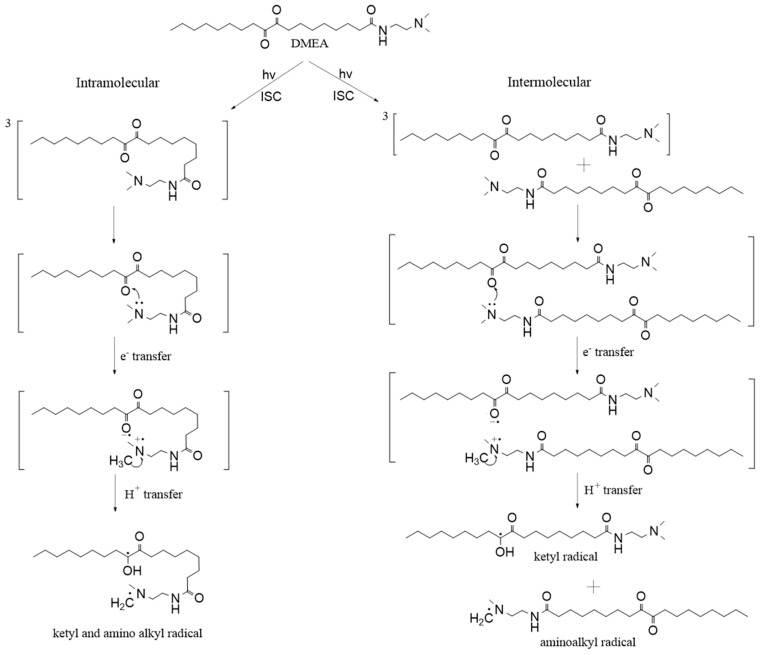
Proposed mechanism for formation of radicals upon excitation of DMEA due to inter- or intra-molecular charge transfer (from the lone pair of electrons on the tertiary amine group to the α-diketo group), showing the initial electron-transfer step.

Malval et al. reported a high rate constant (~10^8^–10^9^ M^−1^s^−1^) for interactions between TEA and α-diketo groups of (1-phenyl-1,2-propanedione, benzil, 9,10-phenanthrenequinone, acenaphthenequinone or dibenzo[a,d]cyclohepta [1,4]-diene-10,11-dione) in acetonitrile upon excitation at 355 nm [51]. Excitation of the α-diketo group to its n-ᴫ* singlet state is followed by intersystem crossing (ISC) to the triplet excited state [52]. In the presence of a tertiary amine group, an electron transfer can occur [53]. Subsequent proton transfer from the tertiary amine cation-radical to the α-diketo anion-radical results in an amino-alkyl radical and a ketyl radical [54]. The corresponding probable interactions between α-diketo and tertiary amine groups in DMEA are shown in Scheme 2. Furthermore, the charge-transfer process between a tertiary amine and an α-diketo group in the solution can be inter- or intra-molecular due to conformational flexibility (Scheme 1). The overall process is slow, occurring over periods of days, in the dark or with radiation from our fluorimeter lamp.

**Figure 9:**
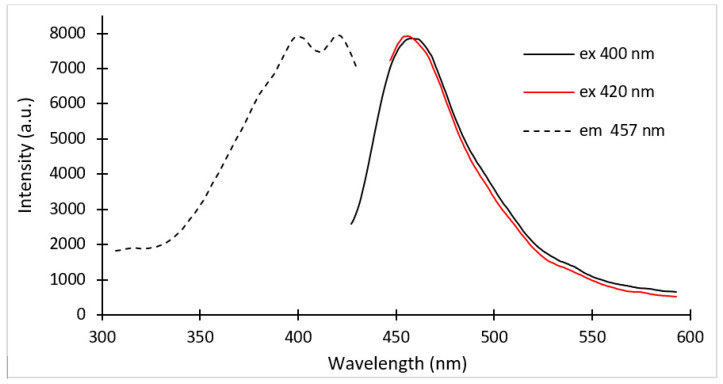
Fluorescence spectra of 3 wt% DIPA in silicone oil at room temperature in a 2 mm (optical path) flattened pyrex capillary. Emission wavelengths (λ_em_ = 457 nm; black, dotted line) and excitation wavelengths (λ_ex_ = 400 nm; black, solid line and 420 nm; red, solid line). The capillary was placed at a 45° angle with respect to the incident beam and the detector direction (transmission).

**Figure 10:**
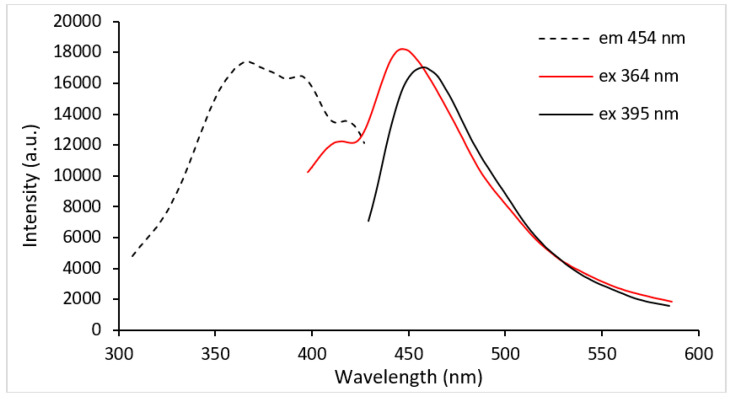
Fluorescence spectra of 3 wt% DMEA in silicone oil at room temperature in a 2 mm (optical path) flattened pyrex capillary. Emission wavelengths (λ_em_ = 454 nm; black, dotted line) and excitation wavelengths (λ_ex_ = 364 nm; red, solid line and 395 nm; black solid line). The capillary was placed at a 45° angle with respect to the incident beam and the detector direction (transmission).

Photophysical properties of 3 wt% DIPA and DMEA gels in silicone oil were explored, as well. The excitation spectrum (λ_em_ = 457 nm) for the DIPA gel showed a shoulder at ~381 nm with two maxima at ~400 and ~420 nm (Figure 9). The excitation spectrum (λ_em_ = 454 nm) of the DMEA gel showed a broad emission maximum centered at ~364 nm with two other peaks of comparable intensity at ~395 nm and ~420 nm, and a shoulder at ~381 nm (Figure 10). The DMEA gel also showed a peak at ~364 nm, which was absent in the DIPA gels. On day 2 after sample preparation, the excitation spectrum for DMEA gels (λ_em_ = 454 nm) displayed a peak at ~364 nm which was already more intense than the peaks at 395 nm and 419 nm. On day 15 after sample preparation, only one peak, centered at ~364 nm, was observed, and the peaks at 395 nm and 419 nm had disappeared (Figure S37), indicating complete conversion of the α-diketo group to a ketyl radical. Thus, even in the aggregated state, the lone pair of electrons from the tertiary amine group of DMEA has a discernible effect on the photophysical properties of the α-diketo group (Scheme 2).

As mentioned above, inter- or intra-molecular charge transfer is not feasible in that fraction of the DMEA molecules that (at any moment) is a part of the gelator network. However, a small fraction of the DMEA molecules, corresponding to approximately the CGC, remains free to migrate as it would in the sol or solution phase [55]. The CGC for DMEA in silicone oil is 0.6 wt%, while the concentration used for photophysical studies is 3 wt%. Conformational changes between the extended molecules in the gelator network and molecules in the sol/solution part of the gel allow the necessary proximity between the tertiary amine and α-diketo groups for the initial electron transfer that is necessary to initiate the changes outlined in Scheme 2.

To determine whether the electron transfer process can be reversed thermally, a freshly prepared 3 wt% DMEA in silicone oil gel was converted to a sol by heating to 75 °C (see DSC thermogram in Figure 2f). At that temperature. Only one broad peak centered at ~364 nm was discernible (Figure S38). Upon reforming the gel (at 0 °C and 25 °C), a broad maximum centered at ~364 nm was observed, along with a shoulder at 395 nm and a peak at 419 nm. Thus, reproducibility was not observed, the peak intensities at 395 nm and 419 nm were reduced significantly compared to the peak at ~364 nm, and changes to the α-diketo group in DMEA are thermally irreversible.

## 4. Conclusions

The single molecule and aggregate properties of three structurally related gelators, DIBA, DIPA, and DMEA, were examined, especially for their ability to form gels in different organic liquids. The gel structures are fibrillar, some in spherulitic bundles. Transmission electron microscopy images of the gelator aggregates show that some of the fiber bundles are twisted. Each molecule is a secondary amide and contains and octadecyl chain and an α-diketo group at the 9,10 positions. Due to the different groups attached to the amide nitrogen atom, the ability of each of the three gelators to form gels differs, and some of the gels exhibit thixotropic behavior which may be important in future applications. However, all packs in conformationally extended lamellar arrangements within their neat and gelled phases.

An important aspect of this research is the step-by-step development of deductive arguments leading to the attribution of the temporal instability of DMEA. As the molecular end of the amide group of DMEA includes a tertiary amine (with a lone pair of electrons), it was found to undergo unimolecular and aggregate electron transfer processes (depending on its concentration and phase) that lead to its reactivity in solution/sol and gel states. Results from powder X-ray diffraction explain why neat DMEA in its crystalline state is both thermally and photochemically stable—the tertiary amine and diketo sites are not near each other and cannot diffuse into proximity. By contrast, the structural analog of DMEA, with an isopentyl group (DIPA), lacks a tertiary amine group (and a lone pair of electrons!) along the amide chain and is more stable to heat and light in its solution/sol and gel phases. However, when a tertiary amine, triethylamine, was added, DIPA became unstable (such as DMEA) due to *inter*molecular electron transfer processes.

## Figures and Tables

**Table 1:** Sample appearances of 2 wt% DIBA, DIPA, and DMEA in various liquids ^a^ and melting point ranges (mps), and critical gelator concentrations (CGCs) of the 2 wt% DIBA samples.

Liquid	DIBA (mp, CGC)	DIPA	DMEA
1-butanol	Soln	-	Soln
acetonitrile	ppt	WG	ppt
benzene	Soln	-	Soln
carbon tetrachloride	WG	Soln	WG
chlorobenzene	-	Soln	-
cyclohexane	-	WG	WG
decane	OG (67–74 °C, >1 wt%) ^b^	OG	-
dimethyl sulfoxide	WG	WG	WG
hexylbenzene	CG (45–52 °C, >1 wt%) ^b^	CG	WG
isostearyl alcohol	-	Soln	-
nitrobenzene	-	Soln	-
octane	OG (68–72 °C, >1 wt%) ^b^	OG	WG
silicone oil DC 550	CG (76–81 °C, 0.6 wt%) ^c^	CG	CG
toluene	WG	Soln	Soln
water	I	I	I
xylene	-	Soln	-

^a^ PPT = precipitate; Soln = solution/sol; WG–weak gel (flowed when the inverted tube was tapped gently); OG = opaque gel (did not flow after tapping the inverted tube); CG = clear gel (did not flow after tapping the inverted tube); I = insoluble during initial heating. ^b^ The CGCs of the octane, decane, and hexylbenzene gels were determined from a series of samples whose concentrations were changed in increments of 1 wt%. All formed inhomogeneous solutions/sols at 1 wt% and gels at 2 wt%. ^c^ CGCs determined from a series of samples whose concentrations were changed in increments of 0.2 wt%.

**Table 2:** Stabilities ^a^ at room temperature of gels of 2 wt% gelators in various liquids.

Liquid	DIBA	DIPA	DMEA
dimethyl sulfoxide	1w	1w	2w
hexylbenzene	<1w	<1w	<1w
octane	3w	3w	5w
silicone oil	8w	8w	6w

^a^ w = week; period during which gels did not fall under the force of gravity when inverted or did not phase-separate.

**Table 3:** Gel-to-sol transition temperatures (°C) from melting of 3 wt% silicone oil gels in a vial and from the peak maxima from DSC thermograms (4 cycles) of the gels.

Gelator	Gel-to-Sol Transition Temperatures (°C)				
	From 4 DSC Heating Cycles				From Heating Gel in a Vial ^a^
	I	II	III	IV	
DIBA	93.1	89.7	88.7	88.1	83.0–87.0
DIPA	84.5	84.9	84.5	84.5	80.0–86.0
DMEA	68.2	66.5	65.8	65.1	59.0–65.0

^a^ Melting points of gels were recorded by immersing inverted glass tubes with the gels into a water bath while heating at a rate of 1–2 °C/min; the temperature ranges show where the gels started to fall and the point where they were completely converted to sols that fell under the influence of gravity.

**Table 4:** Melting temperatures (°C) of neat gelators at maximum heat flows as determined by DSC (4 cycles) and on the hot stage of a microscope.

Gelator	Melting Temperature (°C)				
	Heating Cycles (DSC)				Melting Point ^a^
	I	II	III	IV	
DIBA	99.4	96.9	96.4	96.1	99.1–101.2
DIPA	95.0	92.7	92.4	92.2	93.3–95.6
DMEA	88.3	83.2	80.1	77.0	84.5–85.3

^a^ Melting point ranges of neat gelators recorded on the microscope at a heating rate of ~1 °C/min.

**Table 5:** Bragg diffraction angles (2θ), interplanar spacings (*d*), line widths at half the maximum intensities (FWHM) and calculated mean sizes of the ordered (crystalline) domains (L) for gels with 7 wt% gelator in silicone oil. The L values were calculated using the strongest intensity peaks, at θ ~2° and ~21°.

Gelator	2θ (°)	*d* (Å)	FWHM (°)	L (Å)
DIBA	2.74	32.22	0.60	135
	21.59	4.11	0.52	159
DIPA	2.69	32.82	0.44	187
	21.60	4.11	0.44	174
DMEA	2.59	34.48	0.32	247
	21.38	4.15	0.32	250

## Supplementary Materials

The following supporting information can be downloaded at: https://www.mdpi.com/article/10.3390/gels9010036/s1, 1H and 13C NMR spectra, Infrared spectra, Procedures (gelation studies, thermogravimetric analyses, polarizing optical micrographs, transmission electron micrographs, powder X-ray diffraction, rheology), Photophysical studies. References [16,56,57] are cited in the supplementary materials.
