# Supplementary material for: Molecular and Aggregate Structural, Thermal, Mechanical and Photophysical Properties of Long-Chain Amide Gelators Containing an α-Diketo Group in the Presence or Absence of a Tertiary Amine Group
Source: Gels. 2022 Dec 31;9(1):36. doi: 10.3390/gels9010036 (PMC9857964; doi:10.3390/gels9010036)
Supplement: Supplementary file 1 [file gels-09-00036-s001.zip › gels-2109696-supplementary.pdf]

Supplementary Information

Molecular and Aggregate Structural, Thermal, Mechanical and Photophysical Properties of Long-Chain Amide Gelators Containing an α-Diketo Group in the Presence or Absence of a Tertiary Amine Group

Girishma Grover[1], Andrea Blake Brothers[2] and Richard G. Weiss[1,*]

[1] Department of Chemistry and Institute for Soft Matter Synthesis and Metrology, Georgetown University, Washington, DC 20057, USA
[2] Department of Chemistry, American University, Washington, DC 20016, USA
* Correspondence: weissr@georgetown.edu

Table of content

Syntheses 1
1.1. DODEE (9,10-dioxooctadecanoic acid ethyl ester) 1
1.2. DODA (9,10-dioxooctadecanoic acid) 1
1.3. DIBA (N-isobutyl-9,10-dioxooctadecanamide) 2
1.4. DIPA (N-isopentyl-9,10-dioxooctadecanamide) 2
1.5. DMEA (N-(2-(dimethylamino)ethyl))-9,10-dioxooctadecanamide) 2
^{1}H and ^{13}C NMR spectra 3
Infrared spectra 7
Gelation studies 9
Thermogravimetric analyses 10
Polarizing optical micrographs 10
Transmission Electron Microscopy 11
Powder X-ray diffraction 15
Rheology 17
Photophysical studies 21

1. Syntheses

DODEE (9,10-dioxooctadecanoic acid ethyl ester):

The procedure for synthesis of DODEE has been reported [1]. A solution of ethyl oleate (20.0 mL, 56.2 mmol) was dissolved in 120 mL of acetic anhydride in a round-bottom flask and stirred for 72 h under a nitrogen atmosphere at room temperature. The solution remained colorless. The round-bottom flask was placed in an ice-salt bath, cooled to ~ -10 to -12 °C, and potassium permanganate (11.0 g, 69.6 mmol) was slowly added under a nitrogen atmosphere while the reaction temperature was maintained < -6 °C. After ~ 0.5 h, an additional 13.0 g (82.3 mmol) potassium permanganate was slowly added to the reaction mixture under a nitrogen atmosphere and the temperature was allowed to rise slowly to -3 °C and after another 0.5 h, , and another aliquot of potassium permanganate (12.0 g, 75.9 mmol) was slowly added to reaction mixture while the temperature rose slowly to 10 °C. Then, an ice-cold mixture of 100 mL of saturated aqueous NaCl and 100 mL of aqueous saturated $NaHSO_3$ were added to the reaction mixture, followed by the addition of 100 mL of ethyl acetate and 50 mL of hexane. After stirring the mixture for 20 min, the organic layer was separated and washed sequentially with an ice-cold mixture of 50 mL of saturated aqueous NaCl and 50 mL of aqueous saturated $NaHSO_3$, 4 times with 50 mL of an aqueous saturated $NaHCO_3$, and twice with 50 mL of deionized water. The organic layer was dried over Na_2SO_4 and filtered, and excess ethyl acetate and hexane were removed at 50 °C on a rotary evaporator to obtain a yellow solid which was recrystallized from 10 mL of methanol to obtain 6.1 g (32 % yield) of DODEE as yellow, crystalline needles, mp 42.2- 43.6 °C. ^{1}H NMR ($CDCl_3$, 400 MHz) δ (ppm) (Figure S1): 0.86-0.89 (t, 3 H, -C**H**$_3$, J = 6.9 Hz), 1.24-1.31 (m, 19 H, -C**H**$_2$-C**H**$_2$-, -OCH$_2$C**H**$_3$), 1.53-1.63 (m, 9 H, -C**H**$_2$CH$_2$(C(=O))$_2$, -C**H**$_2$-CH$_2$-C=O, **H**$_2$O), 2.26-2.30 (t, 2 H, C**H**$_2$-C=O, J = 7.5 Hz), 2.70-2.74 (t, 4 H, -C**H**$_2$(C(=O))$_2$-, J = 7.4 Hz), 4.0-4.15 (q, 2 H, -OC**H**$_2$CH$_3$, J = 7.1 Hz). IR (neat solid, cm^{-1}) (Figure S9): 2923 cm^{-1} (alkyl, C-H stretching), 1737 cm^{-1} (ester, C=O stretching) and 1714 cm^{-1} (α-diketo C=O stretching [2]).

DODA (9,10-dioxooctadecanoic acid):

The procedure for synthesis of DODA is the same as reported previously [1]. In a round-bottom flask, DODEE (6.0 g, 17.6 mmol) was dissolved in a mixture of 90 mL of 4% sulfuric acid in acetic acid and refluxed for 2 h. The concentrate was cooled and diluted with 100 mL of water. After vacuum filtration and washing the solid thrice with 15 mL aliquots of methanol, the solid was recrystallized in 20 mL of hexane to obtain 3.0 g of a golden solid crystal (55 % yield), mp 84.0-84.9 °C (lit [1]: 84.1-84.8 °C). ^{1}H NMR ($CDCl_3$, 400 MHz) δ (ppm) (Figure S2): 0.86-0.89 (t, 3 H, -**C*H*$_3$**, J = 6.8 Hz), 1.26-1.36 (m, 16 H, -C**H**$_2$-C**H**$_2$-), 1.54-1.65 (m, 6 H, -C**H**$_2$CH$_2$(C(=O))$_2$ and -C**H**$_2$-CH$_2$-COOH), 2.33-2.37 (t, 2 H, C**H**$_2$-COOH, J = 7.5 Hz), 2.71-2.74 (t, 4 H, -C**H**$_2$(C(=O))$_2$, J = 7.4 Hz). IR (neat solid, cm^{-1}) (Figure S10): 2600-3500 cm^{-1} (carboxylic acid, O-H stretch, broad), 2917 cm^{-1} (alkyl, C-H stretching), 1712 cm^{-1} (α-diketo C=O stretching [2]), 1689 cm^{-1} (carboxylic acid, C=O stretching).

DIBA (N-isobutyl-9,10-dioxooctadecanamide) [3]:

In a two-neck round-bottom flask, 15 mL dry THF was mixed with ethyl chloroformate (0.9 mL, 9.6 mmol), and cooled to 0 °C under nitrogen. An ice-cold mixture of 15 mL dry THF, DODA (1.0 g, 3.2 mmol) and triethylamine (2.2 mL, 12.8 mmol) was mixed and kept in ice for 1 h and then added dropwise with stirring to the THF-ethyl chloroformate solution over 20 min and stirred for another 30 minutes at 0 °C. Isobutylamine (1.3 mL, 12.8 mmol) in 15mL dry THF that had been cooled to 0 °C was added dropwise with stirring over 30 min. The reaction mixture was stirred for 24 h at room temperature under a nitrogen atmosphere and evaporated to an oily residue on a rotary evaporator. Then, 15 mL ethyl acetate and 15 mL hexane were added to the residue which was washed sequentially with 3M HCl (3 x 10 mL), aq. Na_2CO_3 (3 x 10 mL) and distilled H_2O (2 X 10 mL). The organic layer was heated to ensure that the material was dissolved. A yellow precipitate that formed over 1-2 days was collected by vacuum filtration and recrystallized from 15 mL ethyl acetate to obtain 700 mg of a yellow-golden solid (59 % yield), mp 99.1 – 101.2 °C. ^{1}H NMR ($CDCl_3$, 400 MHz) δ (ppm) (Figure S3): 0.86-0.92 (m, 9 H, -C**H**$_3$), 1.26-1.32 (m, 16 H, -C**H**$_2$-C**H**$_2$-), 1.55- 1.64 (m, 25 H, (-C**H**$_2$CH$_2$C(=O))$_2$, NHC(=O)C**H**$_2$CH$_2$-, **H**$_2$O), 1.73-1.79 (m, 1 H, NHCH$_2$C**H**-), 2.14-2.18 (t, 2 H, NH C(=O)-C**H**$_2$ -, J = 7.6 Hz), 2.70-2.74 (t, 4 H, (-C**H**$_2$C(=O))$_2$, J = 7.3 Hz), 3.07-3.10 (t, 2 H, NHC**H**$_2$-, J = 6.4 Hz), 5.41 (broad s, 1H, NH). ^{13}C NMR ($CDCl_3$, 100 MHz) δ (ppm) (Figure S6): 200 (2C, -(**C**=O)$_2$-), 173 (1C, -NH**C**(=O)-), 47 (1C, -NHC(=O)-**C**H$_2$-), 20-37 (17 C, -**C**H$_2$-, -**C**H-(**C**H$_3$)$_2$), 14 (1C, -**C**H$_3$). IR (neat solid, cm^{-1}) (Figure S11): 3315 cm^{-1} (amide, N-H stretch), 2921 cm^{-1} (alkyl, C-H stretch), 1711 cm^{-1} (α-diketo C=O stretch [2]), 1644 cm^{-1} (amide, C=O stretch), 1553 cm^{-1} (amide, N-H bending). Elemental analysis (average of three runs): C = 72.19 %, H = 11.75 %, N = 3.80 %; calcd: C = 71.89 %, H = 11.24 %, N = 3.81 %.

DIPA (N-isopentyl-9,10-dioxooctadecanamide) [3]:

In a two-neck round-bottom flask, ethyl chloroformate (1.8 mL, 19.2 mmol) and 15 mL dry THF were cooled to 0 °C under nitrogen. An ice-cold solution of DODA (2.0 g, 6.4 mmol) and triethylamine (3.3 mL, 19.2 mmol) in 15 mL dry THF was kept in ice for 1 h, added dropwise over 20 min, and stirred for another 30 minutes at 0 °C. A solution of isopentylamine (3.0 mL, 25.6 mmol) in 15mL dry THF was cooled to 0 °C and added dropwise over 30 min. After the reaction mixture had stirred for an additional 24 h at room temperature under nitrogen, it was reduced to an oily residue on a rotary evaporator and 15 mL ethyl acetate and 15 mL hexane were added. The organic liquid was washed sequentially with 3M HCl (3 x 15 mL), aq. Na_2CO_3 solution (3 x 15 mL) and distilled H_2O (2 X 20 mL). The organic layer was heated to ensure that the material was dissolved. A yellow precipitate that formed over 1-2 days was collected by vacuum filtration and recrystallized from 30 mL ethyl acetate to obtain 1.42 g (58 % yield) of yellow-golden solid, mp 93.3 – 95.6 °C. ^{1}H NMR ($CDCl_3$, 400 MHz) δ (ppm) (Figure S4): 0.86-0.92 (m, 9 H, -C**H**$_3$), 1.26 -1.32 (m, 16 H, -C**H**$_2$-C**H**$_2$-), 1.36-1.41 (m, 3 H, NHCH$_2$C**H**$_2$C**H**(CH$_3$)$_2$), 1.53- 1.65 (m, 10 H, (-C**H**$_2$CH$_2$C(=O))$_2$, C**H**$_2$-NHC(=O)CH$_2$, **H**$_2$O), 2.12-2.16 (t, 2 H, NHC(=O)-C**H**$_2$-, J = 7.6 Hz), 2.70-2.74 (t, 4 H, (-C**H**$_2$C(=O))$_2$), 3.23-3.28 (t, 2 H, C(=O)NHC**H**$_2$-, J = 5.9 Hz), 5.32 (broad s, 1H, N**H**C(=O)). ^{13}C NMR ($CDCl_3$, 100 MHz) δ (ppm) (Figure S7): 200 (2C, -(**C**=O)$_2$-), 173 (1C, -NH**C**(=O)-), 39 (1C, -NHC(=O)-**C**H$_2$-), 38 (1C, -**C**H$_2$-NH C(=O)-), 23-37 (17 C, -**C**H$_2$-, -**C**H-(**C**H$_3$)$_2$), 14 (1C, -**C**H$_3$). IR (neat solid, cm^{-1}) (Figure S12): 3312 cm^{-1} (amide, N-H stretch), 2921 cm^{-1} (alkyl, C-H stretch), 1711 cm^{-1} (α-diketo C=O stretch [2]), 1644 cm^{-1} (amide, C=O stretch), 1553 cm^{-1} (amide, N-H bending). Elemental analysis (average of three runs): C = 72.16 %, H = 12.08 %, N = 3.61 %, calculated: C = 72.39 %, H = 11.36 %, N = 3.61 %.

DMEA (N-(2-(dimethylamino)ethyl))-9,10-dioxooctadecanamide) [3]:

In a three-neck round-bottom flask, an ice-cold solution of ethyl chloroformate (1.8 mL, 19.2 mmol) in 25 mL dry THF was added dropwise with stirring over 20 min to a solution of triethylamine (3.3 mL, 19.2 mmol) and DODA (2.0 g, 6.4 mmol) in 15 mL dry THF (kept over ice for 1 h) at 0 °C under a nitrogen atmosphere. After the mixture had stirred for an additional 30 min at 0 °C, a solution of cold (0 °C) N,N-dimethyl ethylenediamine (2.8 mL, 25.6 mmol) in 15mL dry THF mixture was added dropwise over 15 min. The reaction mixture was stirred for an additional 20 h under a nitrogen atmosphere at room temperature, filtered to remove a white precipitate, and the yellow filtrate was reduced to 1-2 mL of a brownish-yellow oil on a rotary evaporator. The oil was dissolved in 30 mL ethyl acetate and washed thrice with a 10 mL mixture of satd, aq sodium bicarbonate solution and sodium chloride. The organic layer was dried over anhyd magnesium sulfate, filtered, and reduced to ~5 mL on a heated water bath and cooled to 0 °C. The precipitate thus formed was filtered and recrystallized from acetonitrile to obtain 520 mg (21 % yield) of a yellow solid, mp 84.5-85.3 °C. ^{1}H NMR ($CDCl_3$, 400 MHz) δ (ppm) (Figure S5): 0.86-0.89 (t, 3 H, -C**H**$_3$), 1.26-1.31 (m, 16 H, -C**H**$_2$-C**H**$_2$-), 1.58 (m, 44 H, **H**$_2$O, -C**H**$_2$-CH$_2$-(C(=O))$_2$ and C**H**$_2$-C(=O)NH-), 2.15-2.19 (t, 2 H, C(=O)NH-C**H**$_2$), 2.24 (s, 6 H, N(C**H**$_3$)$_2$), 2.40-2.43 (t, 2 H, -C**H**$_2$-N(CH$_3$)$_2$, J = 5.8 Hz), 2.70-2.74 (t, 4 H, -C**H**$_2$-(C(=O))$_2$), 3.30-3.35 (q, 2 H, -C(=O)NH-C**H**$_2$, J = 5.6 Hz), 6.06 (s, broad, 1 H, C(=O)N**H**). ^{13}C NMR ($CDCl_3$, 100 MHz) δ (ppm) (Figure S8): 200 (2C, -(**C**=O)$_2$-), 173 (1C, -NH**C**(=O)-), 58 (1C, -**C**H$_2$-N(CH$_3$)$_2$-), 45 (2C, CH$_2$-N(**C**H$_3$)$_2$-), 23-37 (15 C, -

CH$_2$-), 14 (1C, -**C**H$_3$). IR (neat solid, cm^{-1}) (Figure S13): 3309 cm^{-1} (amide, N-H stretch), 2921 cm^{-1} (alkyl, C-H stretch), 1710 cm^{-1} (α-diketo C=O stretch [2]), 1641 cm^{-1} (amide, C=O stretch), 1554 cm^{-1} (amide, N-H bending). Elemental analysis- experimental (average of six runs): C = 68.89 %, H = 11.30 %, N = 7.29 %; calculated: C = 69.07 %, H = 11.06 %, N = 7.32 %.

^{1}H and ^{13}C NMR spectra

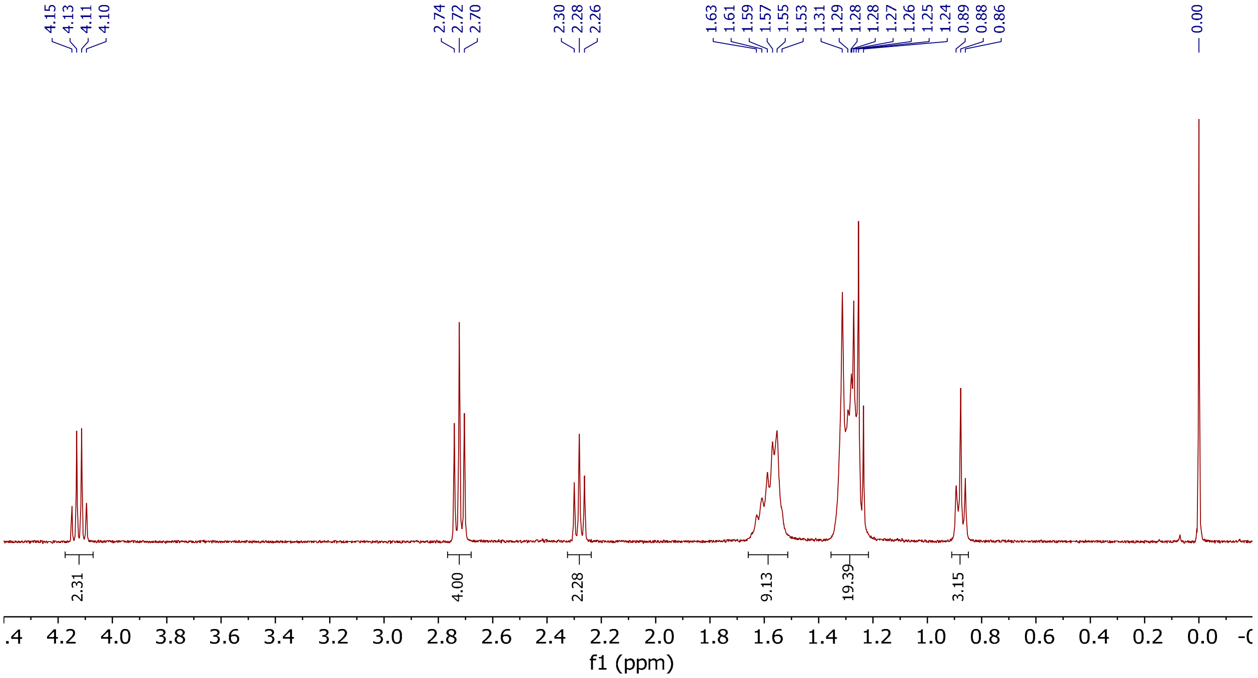

Figure S1. ^{1}H NMR spectrum of DODEE in chloroform-*d*.

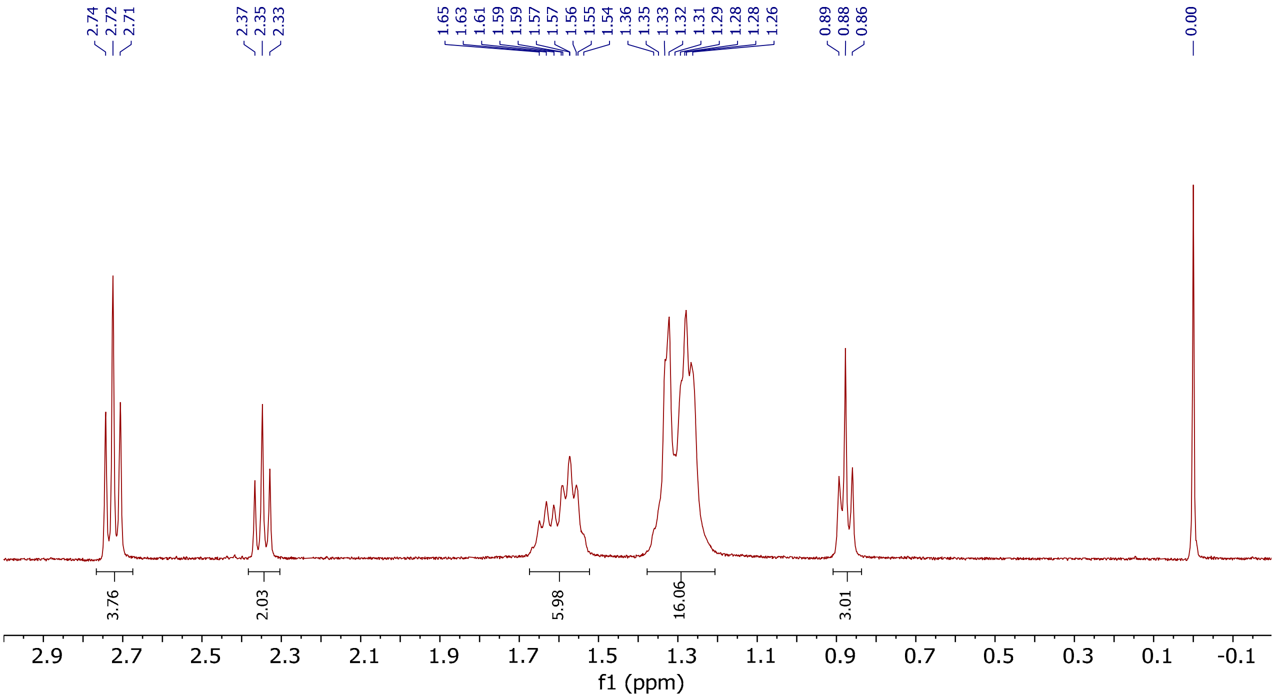

Figure S2. ^{1}H NMR spectrum of DODA in chloroform-*d*.

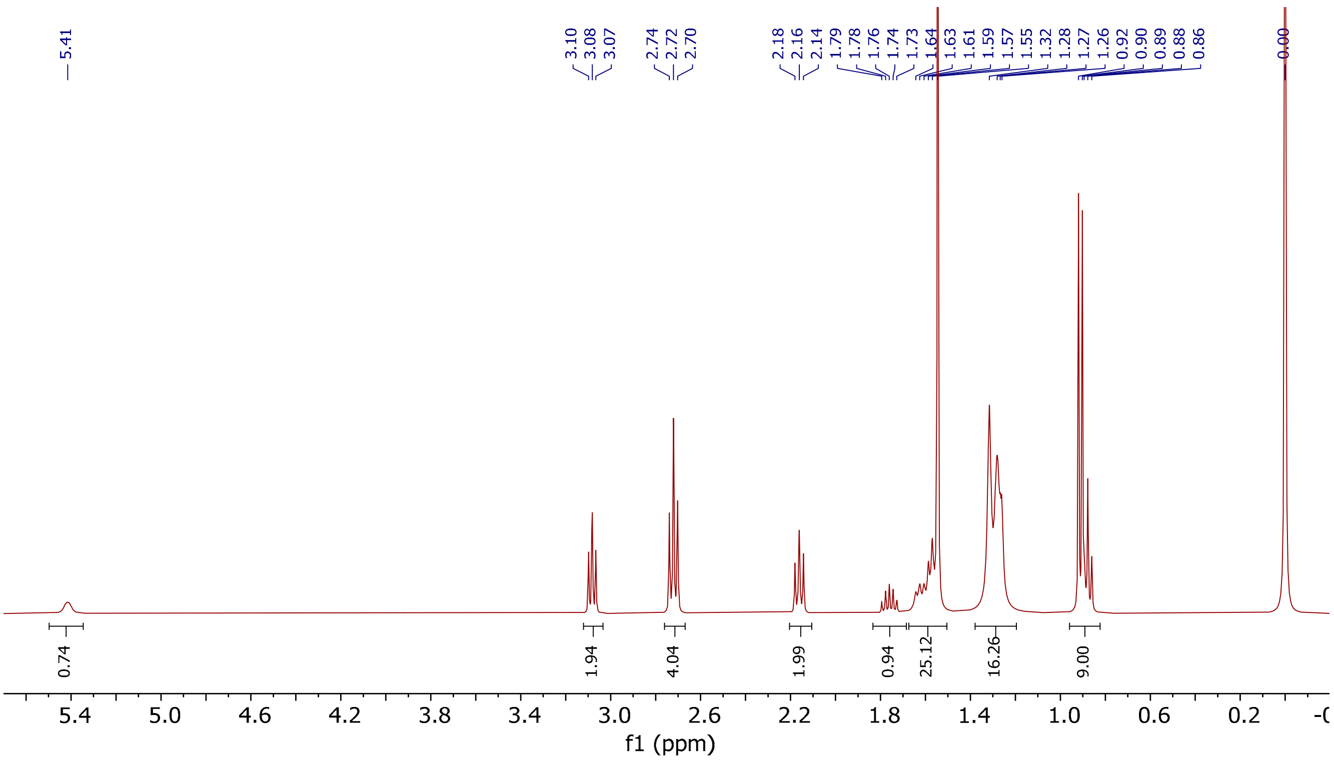

Figure S3. ^{1}H NMR spectrum of DIBA in chloroform-*d*.

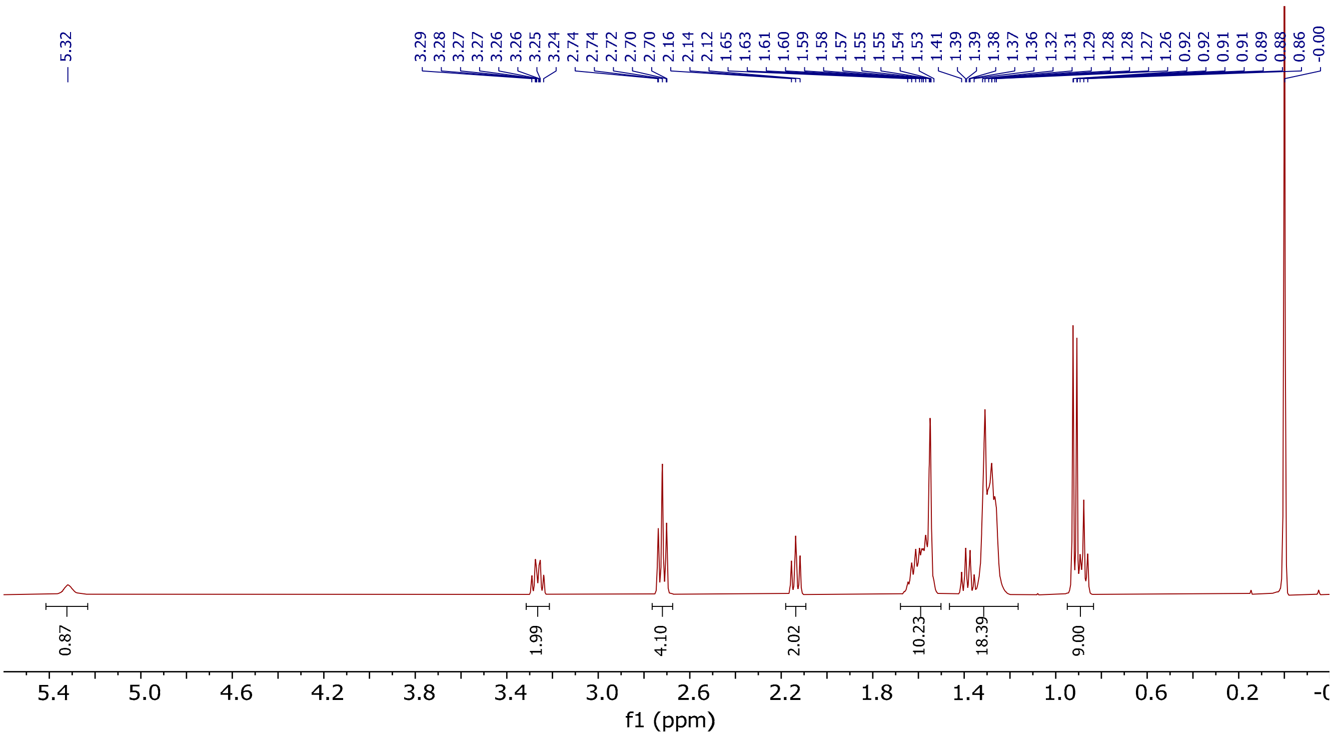

Figure S4. ^{1}H NMR spectrum of DIPA in chloroform-*d*.

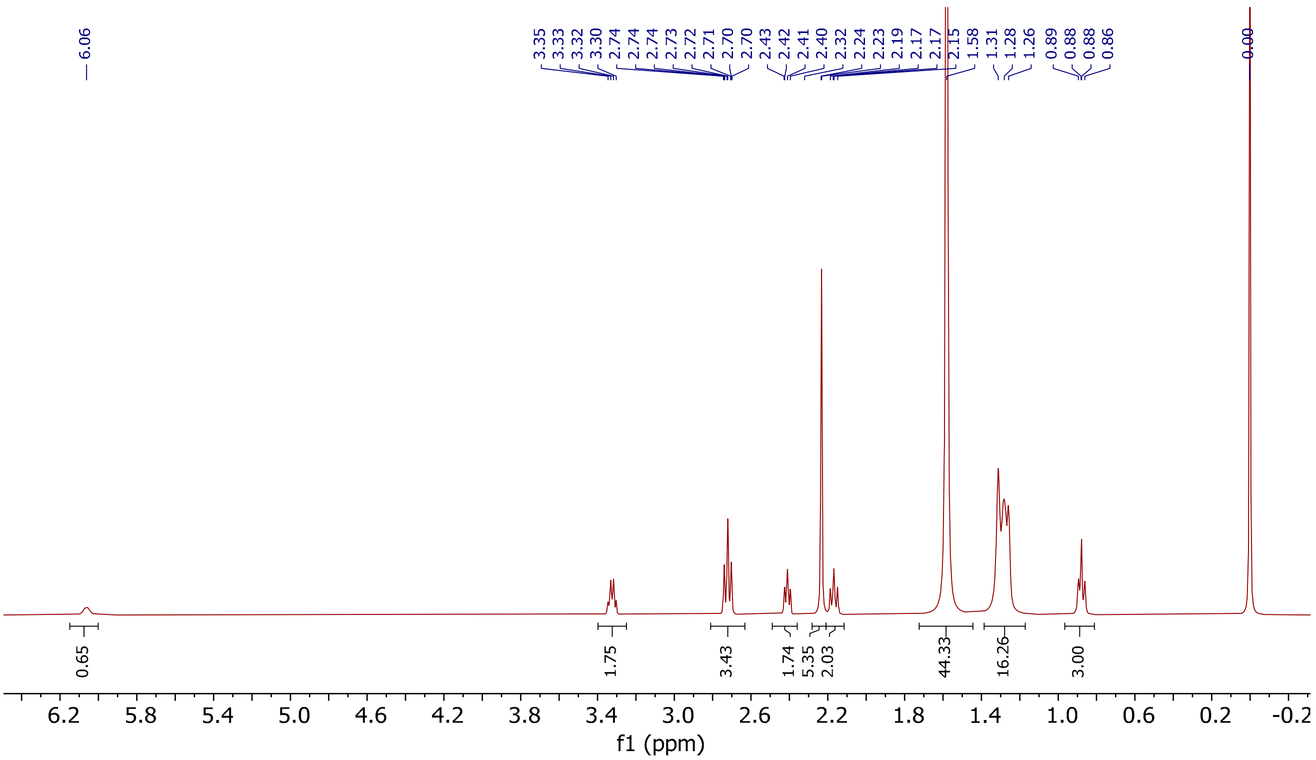

Figure S5. ^{1}H NMR spectrum of DMEA in chloroform-*d*.

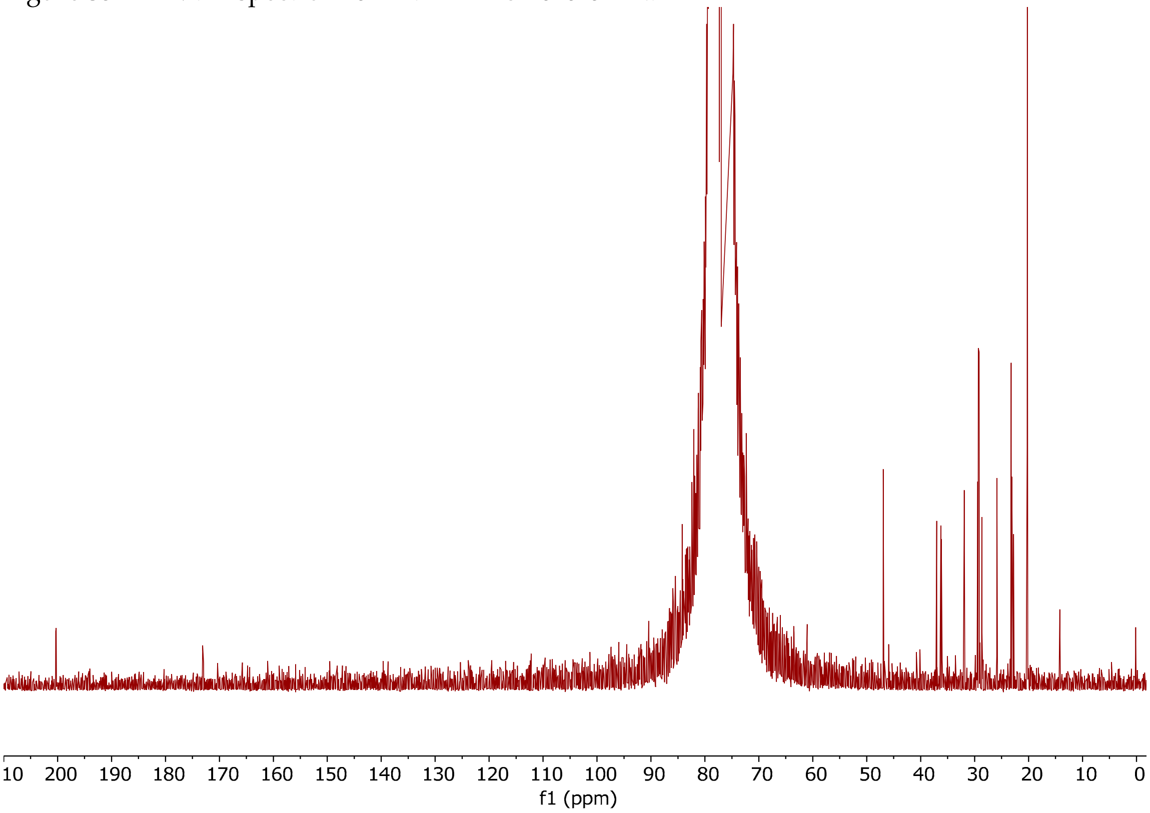

Figure S6. ^{13}C NMR spectrum of DIBA in chloroform-*d*.

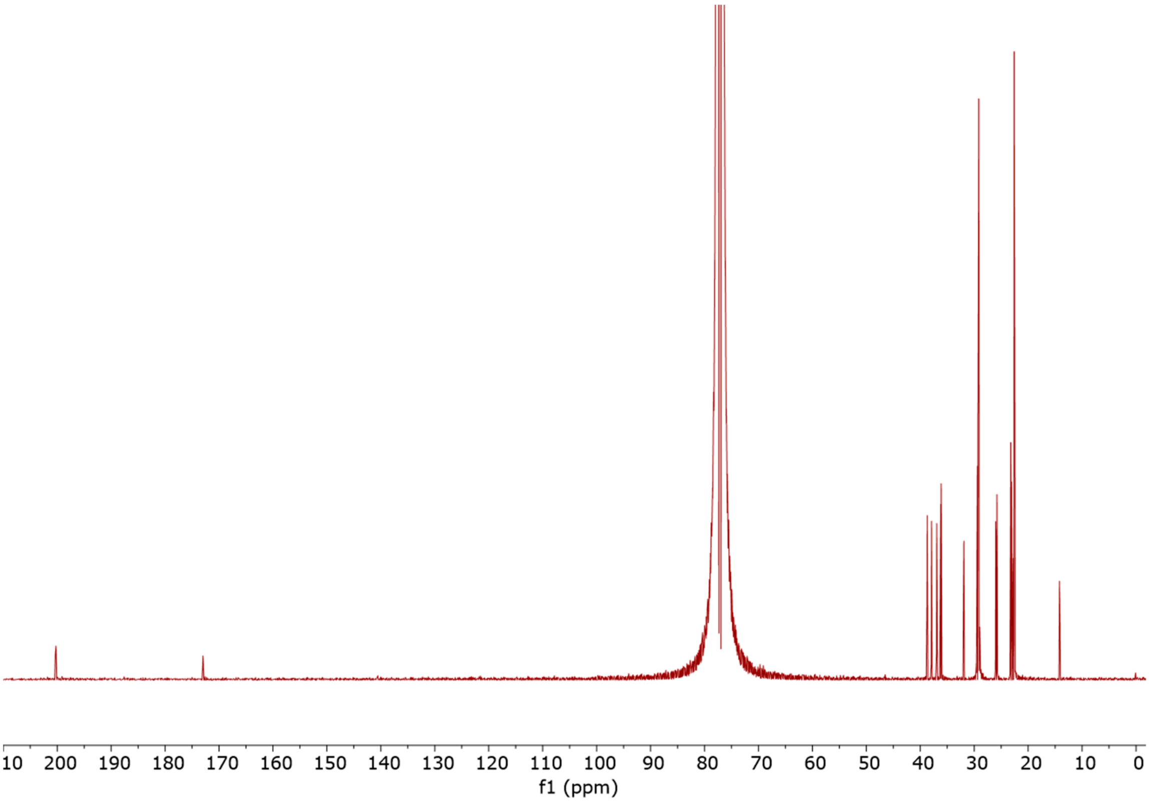

Figure S7. ^{13}C NMR spectrum of DIPA in chloroform-*d*.

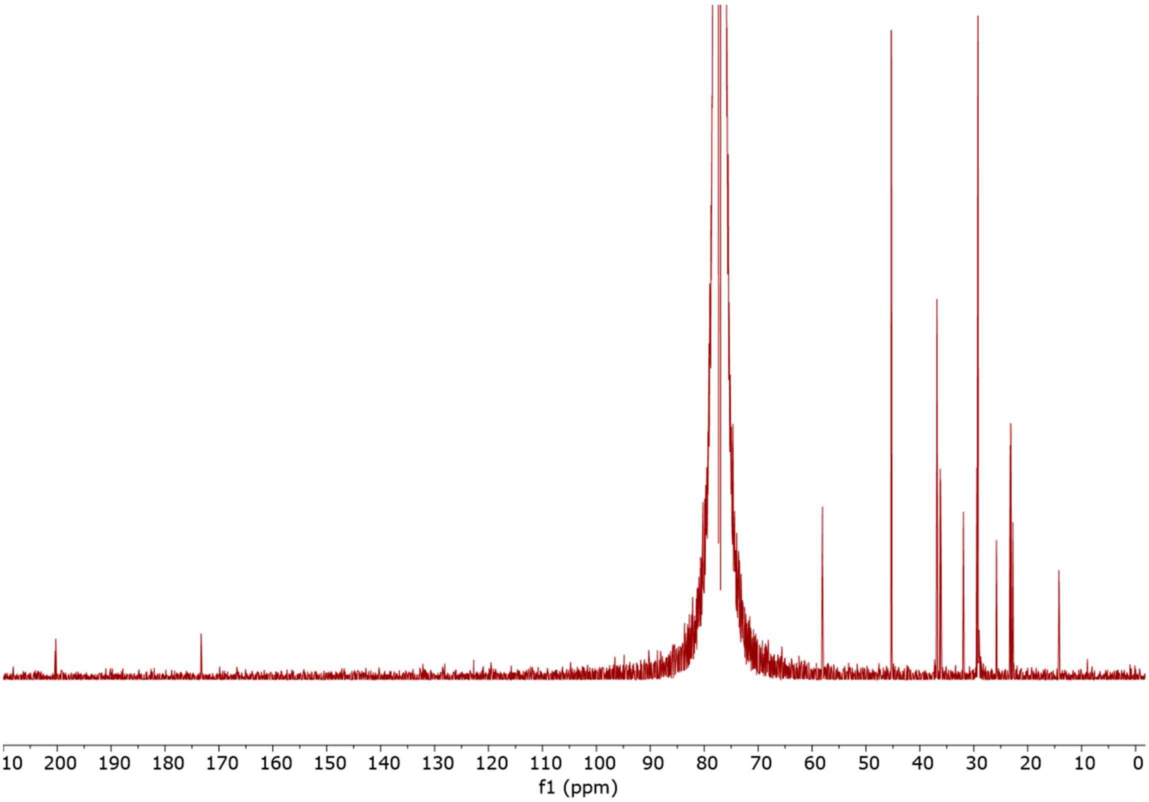

Figure S8. ^{13}C NMR spectrum of DMEA in chloroform-*d*.

Infrared spectra

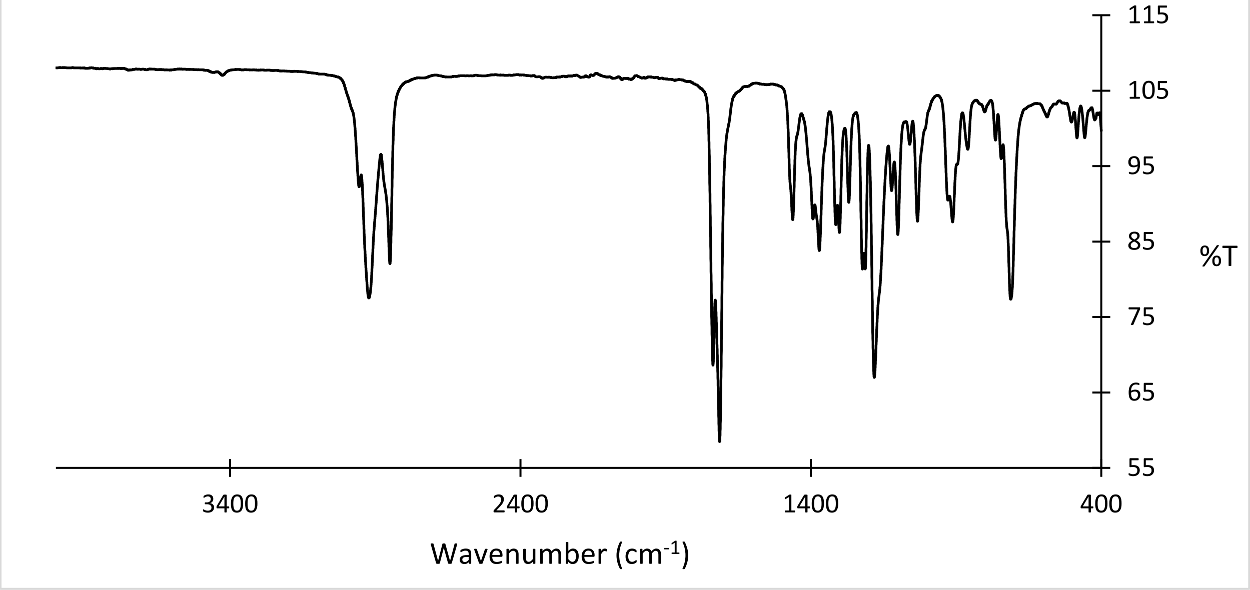

Figure S9. FTIR spectrum of neat DODEE.

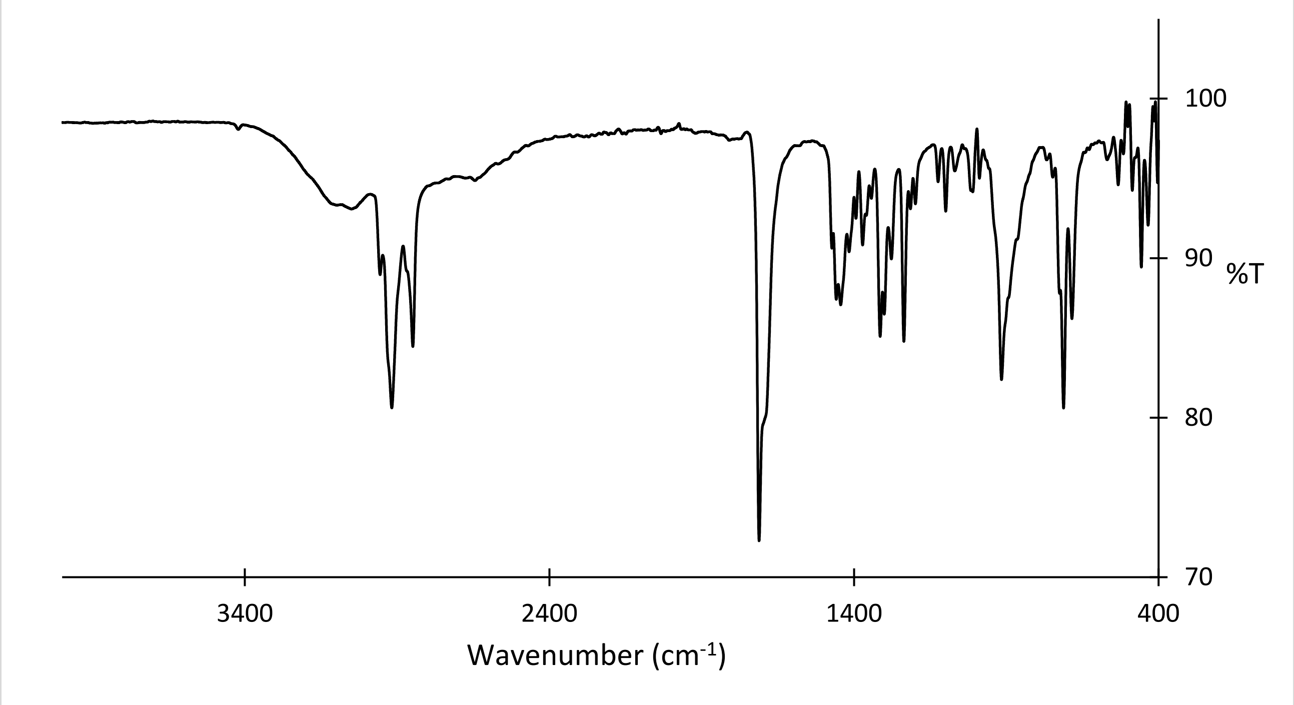

Figure S10. FTIR spectrum of neat DODA.

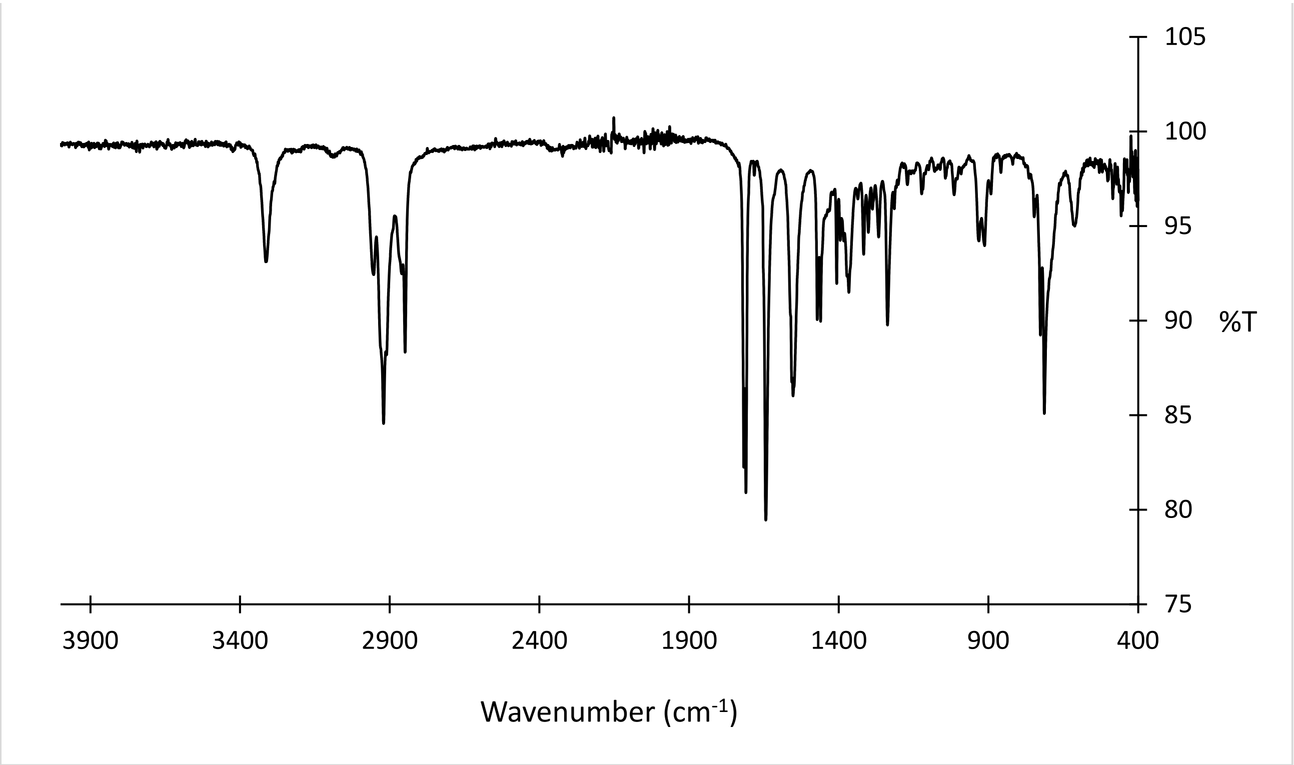

Figure S11. FTIR spectrum of neat DIBA.

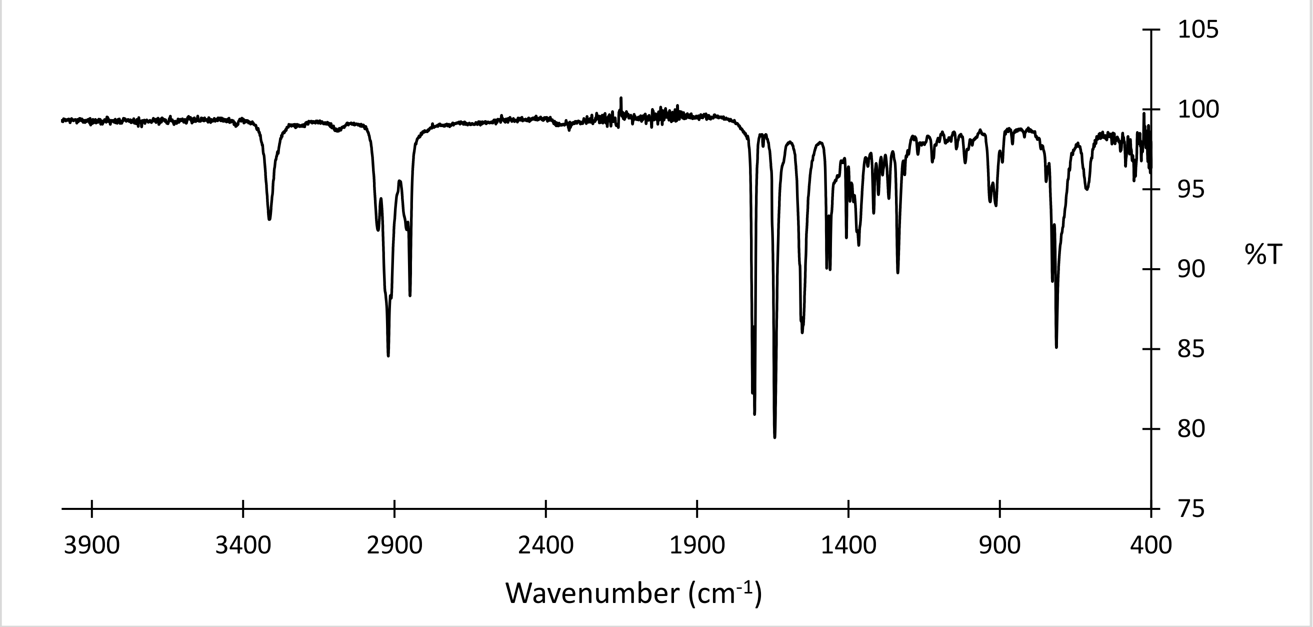

Figure S12. FTIR spectrum of neat DIPA.

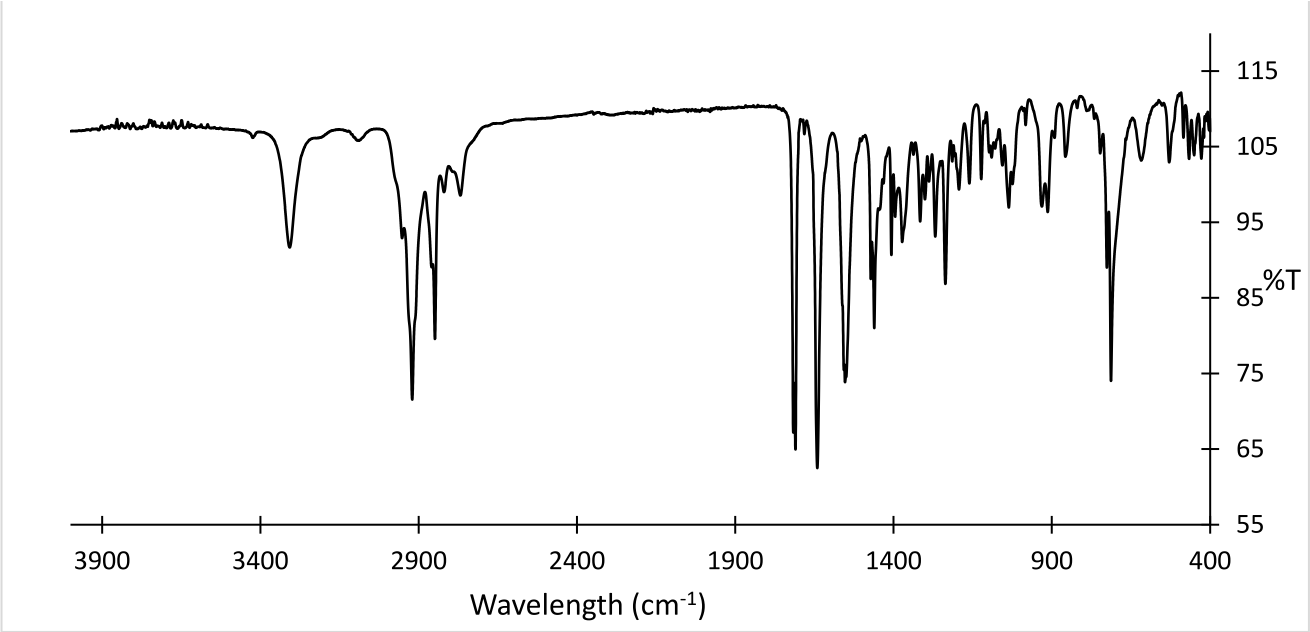

Figure S13. FTIR spectrum of neat DMEA.

Gelation studies

Table S1. Sample appearances[a] of 5 wt % DIBA, DIPA, and DMEA in various liquids and melting point ranges of the DIBA gels (in parentheses).

Liquid	DIBA (mp)	DIPA	DMEA
acetone	PPT	-	-
benzonitrile	Soln	-	-
carbon tetrachloride	Soln	-	WG
chlorobenzene	WG (35-50 ˚C)	-	-
decane	OG (74-89˚C)	-	-
dimethyl sulfoxide	WG (52-60 ˚C)	WG	WG
dimethylformamide	Soln	-	-
hexylbenzene	CG (62-65 ˚C)	-	WG
isostearyl alcohol	-	PPT	-
nitrobenzene	Soln	-	-
octane	OG (78-87 ˚C)	OG	WG
tetrahydrofuran	PPT	-	-
toluene	WG (44-53 ˚C)	-	-
silicone oil DC 550	CG (85-89 ˚C)	CG	CG

[a] PPT = precipitate; Soln = solution/sol; WG – weak gel; OG = opaque gel; CG = clear gel.

Thermogravimetric analyses

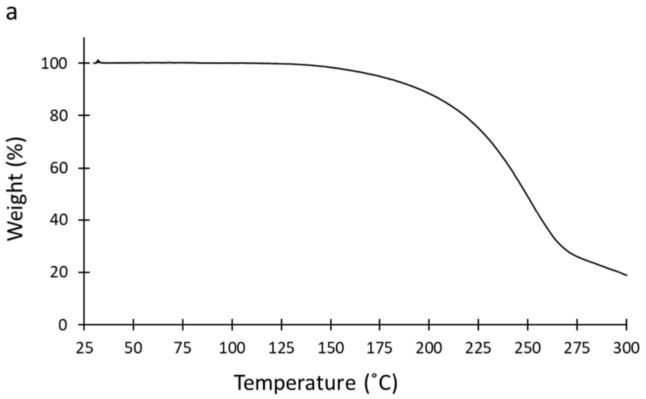

Figure S14. TGA of neat (a) DIBA and (b) DMEA.

According to the thermogravimetric analyses in Figure S14, DIBA is stable to ca. 120 ˚C and DMEA is stable to ca. 100 ˚C.

Polarizing optical micrographs

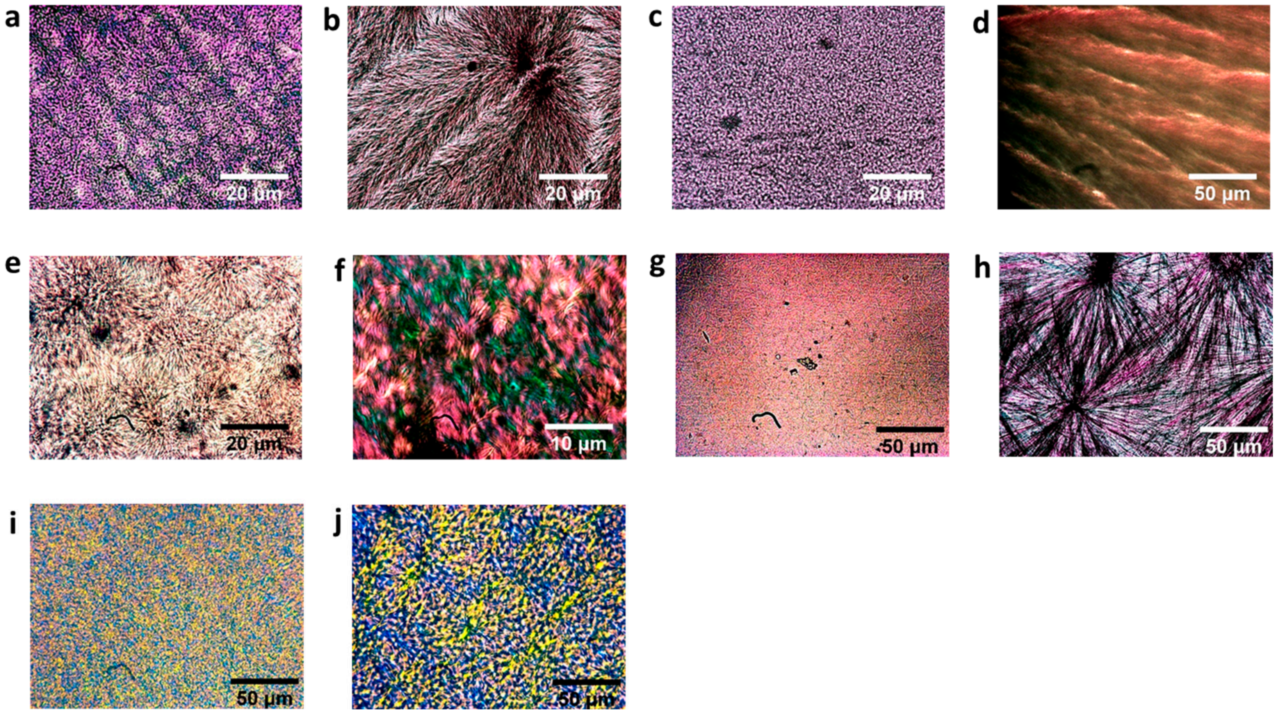

Figure S15. Polarizing optical micrographs at room temperature. 5 wt % DIPA in hexyl benzene gel prepared by (a) fast-cooling method and (b) slow-cooling method. 5 wt % DIPA in octane gel prepared by (c) fast-cooling method and (d) slow-cooling method. 5 wt % DIPA in silicone oil gel prepared by (e) fast-cooling method and (f) slow-cooling method. 5 wt % DIBA in silicone oil gel prepared by (g) fast-cooling method and (h) slow-cooling method. 5 wt % DMEA in silicone oil gel prepared by (i) fast-cooling method and (j) slow-cooling method. The scale bars are: 10 µm (f); 20 µm (a), (b), (c) and (e); 50 µm (d), (g), (h), (i) and (j). DIBA, DIPA and DMEA gels were prepared in flame-sealed, flattened capillary tubes of 2 mm interior thicknesses (silicone oil gels), 1 mm (octane gels), or 0.5 mm (hexylbenzene gels).

Transmission Electron Microscopy

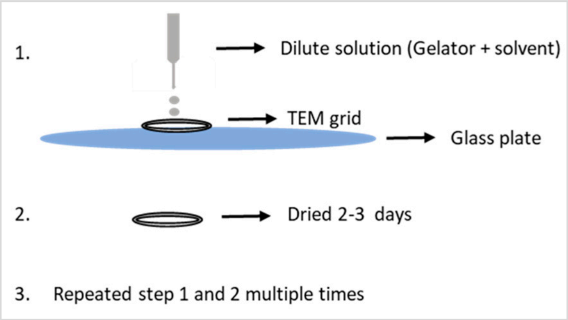

Figure S16. Steps for depositing sol samples on TEM grids.

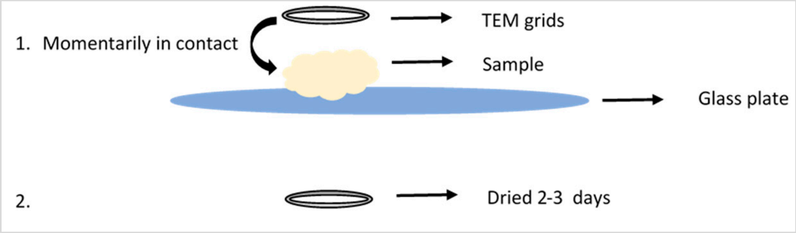

Figure S17. Steps for depositing gel samples on TEM grids.

Note that fiber widths reported in the following tables at the lower magnifications are not as precise as those recorded at higher magnifications. No attempt to determine the differences in precision has been made.

Table S2. Widths of randomly selected individual fibers from the TEM images of 0.05 wt % DIBA in octane at 1k and 2.5k magnifications.

1k magnification	2.5k magnification
Measured fiber width (µm)	Measured fiber width (µm)
0.153	0.165
0.153	0.188
0.153	0.202
0.169	0.209
0.2	0.214
0.223	0.224
0.225	0.307
0.237	0.31
0.25	0.317
0.261	0.381
0.261	0.387
0.261	0.437
0.263	0.533
0.28	0.599
0.32	0.666
0.402	0.691
0.484	0.895
0.643	1.047

0.744	1.085
0.817	1.487

Table S3. Widths of randomly selected individual fibers from the TEM images of 0.5 wt % DIBA in hexylbenzene at 500 and 2.5k magnifications.

500 magnificaton	2.5 k magnification
Measured fiber width (μm)	Measured fiber width (nm)
0.052	44.282
0.078	55.475
0.082	58.899
0.104	63.058
0.11	64.678
0.11	71.425
0.116	74.171
0.13	101.01
0.13	111.111
0.13	121.632
0.13	129.356
0.132	158.106
0.147	171.717
0.166	171.717
0.182	179.843
0.203	180.692
0.214	187.89
0.257	218.051
0.267	266.674
0.281	428.788

Table S4. Widths of randomly selected individual fibers from the TEM images of 2 wt % DIBA in octane at 1k and 10k magnifications.

1k magnification	10k magnification
Measured fiber width (μm)	Measured fiber width (nm)
0.087	130.012
0.11	164.384
0.135	164.579
0.15	199.432

0.178	225.913
0.18	247.321
0.197	251.219
0.198	279.095
0.207	293.319
0.216	296.655
0.221	309.547
0.242	334.308
0.253	343.203
0.261	394.756
0.287	429.712
0.297	438.924
0.407	476.973
0.46	513.235
0.546	576.017
0.565	639.27

Table S5. Widths of randomly selected individual fibers from the TEM images of 2 wt % DIPA in octane at 1k and 6k magnifications.

1k magnification	6k magnification
Measured fiber width(μm)	Measured fiber width(μm)
0.163	0.117
0.167	0.122
0.191	0.14
0.199	0.155
0.216	0.167
0.224	0.17
0.225	0.171
0.236	0.171
0.264	0.175
0.278	0.177
0.304	0.203
0.318	0.211
0.363	0.211
0.381	0.233
0.412	0.233
0.459	0.257
0.529	0.277
0.54	0.285
0.603	0.327
0.609	0.401

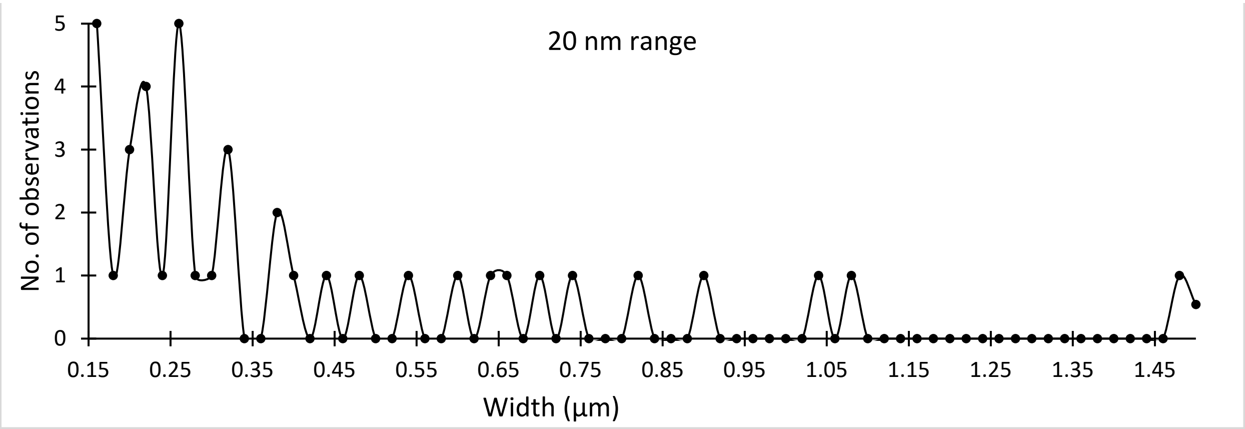

Figure S18. Plot of number of observations lying within a ±0.02 µm range versus fiber width (µm) for 0.05 wt % DIBA in octane. The fiber widths were taken from TEMs at 1k and 2.5k magnifications.

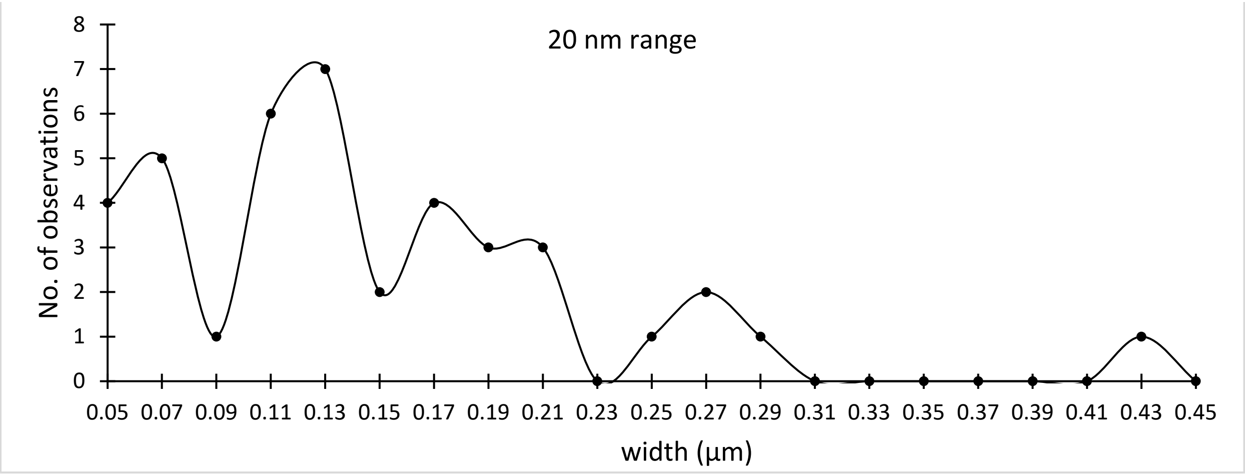

Figure S19. Plot of number of observations lying within a ±0.02 µm range versus fiber width (µm) for 0.5 wt % DIBA in hexylbenzene. The fiber widths were taken from TEMs at 500 and 2.5k magnifications.

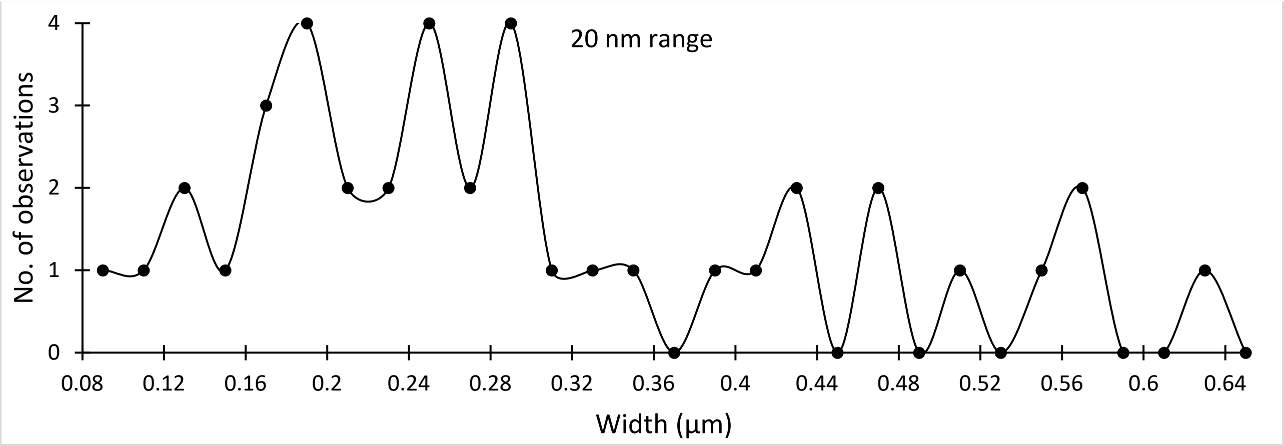

Figure S20. Plot of number of observations lying within a ±0.02 µm range versus fiber width (µm) for 2 wt % DIBA in octane. The fiber widths were taken from TEMs at 1k and 10k magnifications.

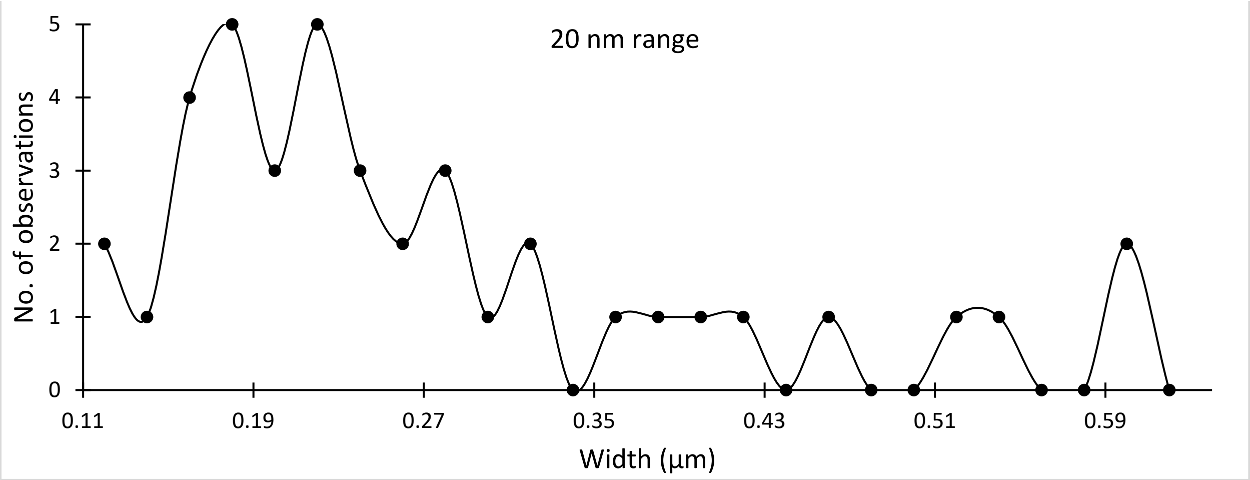

Figure S21. Plot of number of observations lying within a ±0.02 µm range versus fiber width (µm) for 2 wt % DIPA in octane. The fiber widths were taken from TEMs at 1k and 6k magnifications.

Powder X-ray diffraction

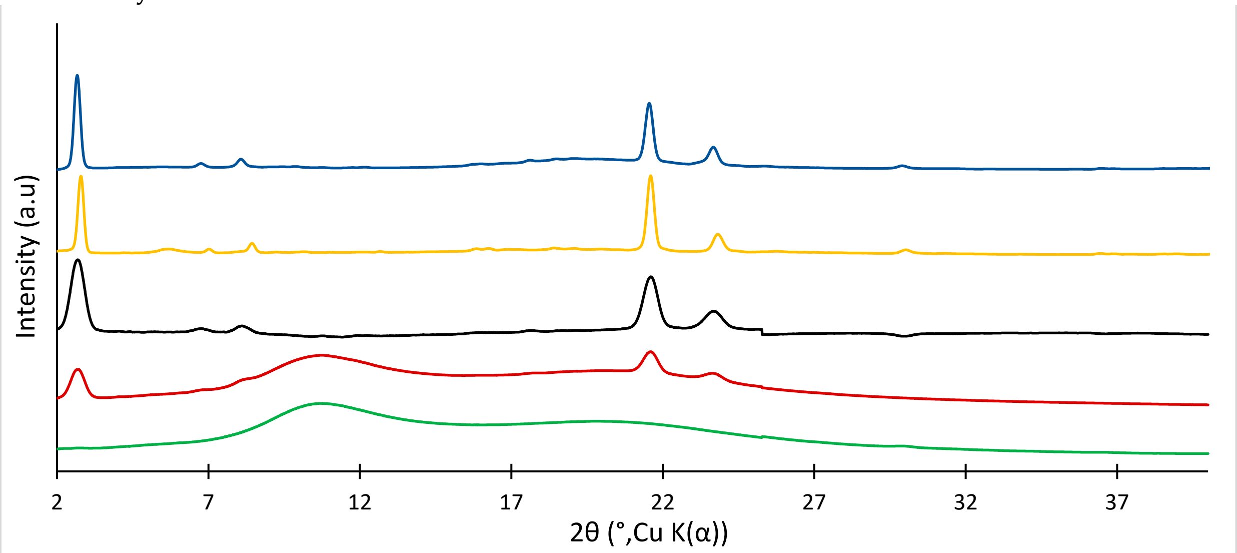

Figure S22. Powder X-ray diffraction patterns: solidified-melt of DIPA (blue); neat-solid DIPA (yellow); empirically subtracted diffraction pattern of silicone oil from (7 wt %)-DIPA in silicone oil (black); (7 wt %)-DIPA in silicone oil (red); silicone oil (green). The Y-axes are offset vertically.

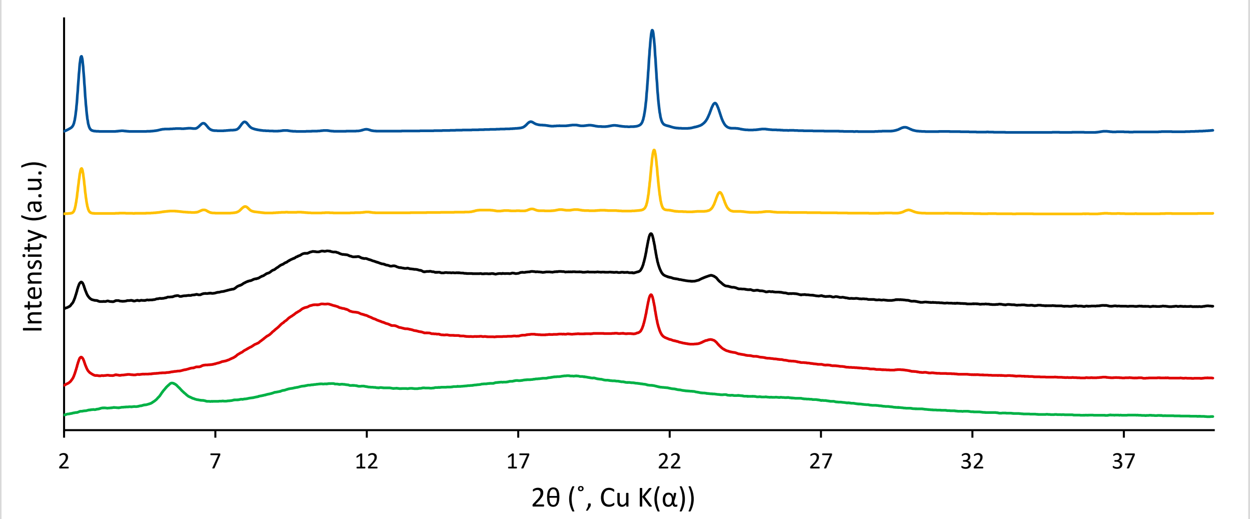

Figure S23. Powder X-ray diffraction patterns: solidified-melt of DMEA (blue); neat-solid DMEA (yellow); empirically subtracted diffraction pattern of silicone oil from (7 wt %)-DMEA in silicone oil (black); (7 wt %)-DMEA in silicone oil (red); silicone oil (green). The Y-axes are offset vertically.

Table S6. Bragg's diffraction angles (2θ) and interplanar spacings (*d*) for a gel of 7 wt % DIBA in silicone oil, neat DIBA and solidified-melt of DIBA.

**DIBA-silicone oil (7 wt %)**		**neat DIBA**		**solidified-melt DIBA**	
**2θ (⁰)**	***d*(Å)**	**2θ (⁰)**	***d*(Å)**	**2θ (⁰)**	***d*(Å)**
2.7	32.2	2.8	**31.7**	2.8	31.1
		5.7	**15.4**		
7.0	12.6	7	12.6		
8.7	10.1	8.4	**10.5**	8.6	10.3
10.8	8.2				
			-	12.2	7.3
				13.5	6.6
				15.2	5.8
		15.8	5.6		
		16.2	**5.5**		
		18.4	4.8		
		19.1	4.6	18.9	4.7
21.6	4.1	21.6	4.1	21.4	4.1
23.8	3.7	23.8	3.7	23.7	3.8
				29.4	3
30	3	30	3	29.9	3
		40.6	2.2	40.4	2.2

Table S7. Bragg's diffraction angles (2θ) and interplanar spacings (*d*) for a gel of 7 wt % DIPA in silicone oil, neat DIPA and solidified-melt of DIPA.

**DIPA-silicone oil (7 wt %)**		**neat DIPA**		**solidified-melt DIPA**	
**2θ (⁰)**	***d*(Å)**	**2θ (⁰)**	***d*(Å)**	**2θ (⁰)**	***d*(Å)**
2.7	32.8	2.6	**33.4**	2.7	33.2
		5.6	**15.7**		
6.8	13			6.8	13.1
8.1	10.9	8	**11.1**	8.1	10.9
10.8	8.2				
		11.9	**7.4**		
		13.3	6.7		
				16	5.5
17.7	5	18	**4.9**	17.6	5
		18.5	4.8		
21.6	4.1	21.6	4.1	21.6	4.1
23.7	3.8	23.8	3.7	23.7	3.8
		25.6	3.5		
30	3	30.1	3	29.9	3
		38.5	2.3		
				40.4	2.2

Table S8. Bragg's diffraction angles (2θ) and interplanar spacings (*d*) for a gel of 7 wt % DMEA in silicone oil, neat DMEA and solidified-melt of DMEA.

**DMEA-silicone oil (7wt%)**		**neat DMEA**		**Solidified-melt DMEA**	
**2θ (⁰)**	***d*(Å)**	**2θ (⁰)**	***d*(Å)**	**2θ(⁰)**	***d*(Å)**
2.6	34.5	2.6	**34.2**	2.6	34.2
		5.6	**15.8**		
		6.6	13.3	6.6	13.4
		8	**11.1**	8	11.1
		9.8	9.1		

10.1	8.8				
		12	**7.4**	12	7.4
		15.9	**5.6**		
17.4	5.1	17.4	5.1	17.4	5.1
		18.9	4.7	18.9	4.7
		19.8	4.5		
21.4	4.2	21.5	4.1	21.4	4.1
23.3	3.8	23.7	3.8	23.5	3.8
		25.2	3.5	25.1	3.5
29.7	3	29.9	3	29.8	3
36.4	2.5	36.4	2.5	36.4	2.5

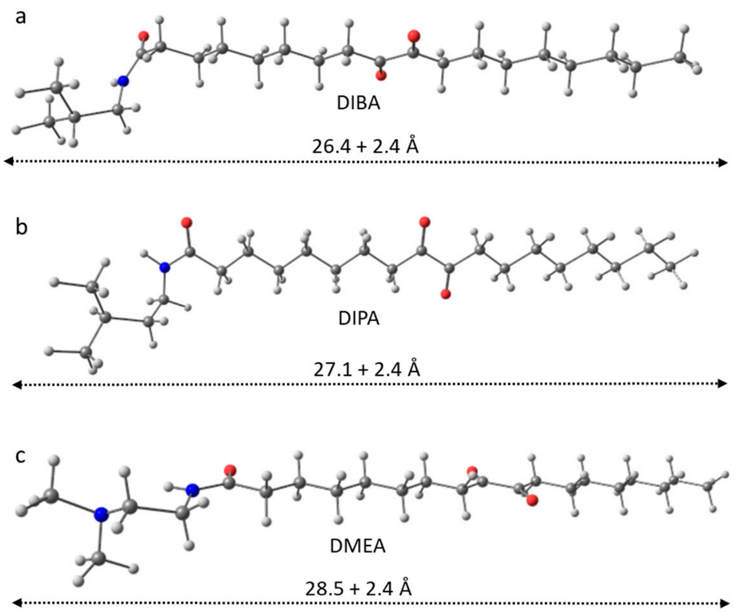

Figure S24. Calculated lengths of gelators from DFT calculations at the B3LYP/6-31G(d,p) level (with the van der Waals radii of hydrogen atoms added at each terminus; 2.4 Å) of energy-minimized extended conformations: a) DIBA (26.4 Å); b) DIPA (27.1 Å); c) DMEA (28.5 Å).

Rheology

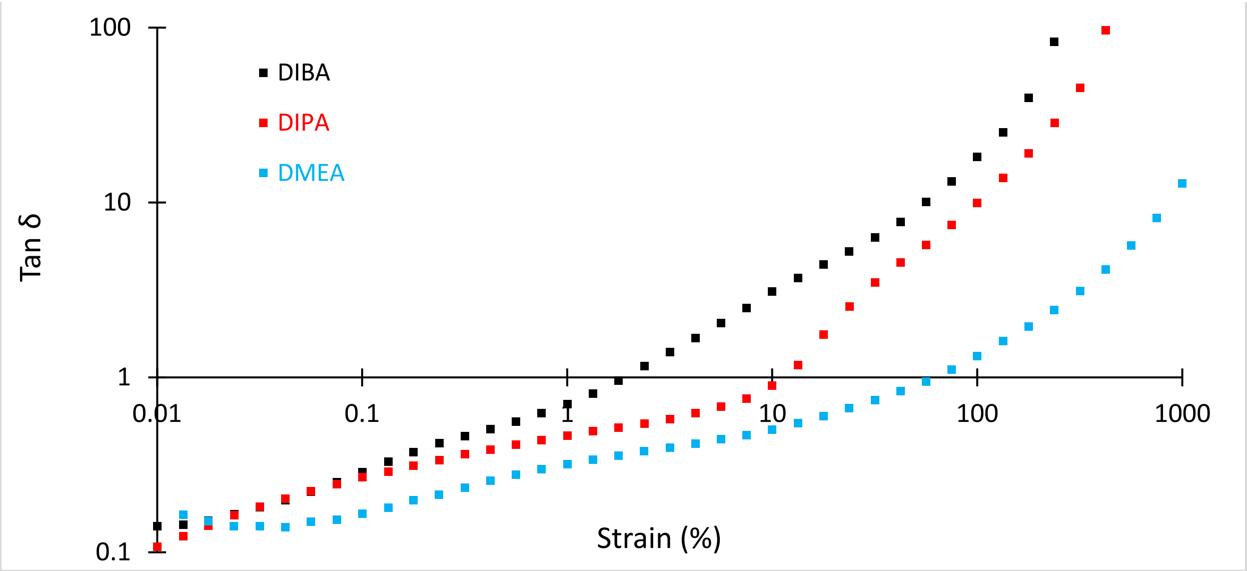

Figure S25. Log-log tan δ versus strain sweeps (1 Hz frequency) at 25 ˚C: 3 wt % DIBA-silicone oil gel (black); 3 wt % DIPA-silicone oil gel (red); 3 wt % DMEA-silicone oil gel (blue).

Table S9. The cross-over points (%) on application of strain for 3 wt % gelators in silicone oil, above which the gels show solid-like behavior and below which the gels show fluid-like behavior.

gel (3 wt %)	cross-over point (strain %)	G' > G" (solid-like) (strain %)	G' < G" (fluid-like) (strain %)
DIBA	2.1 ± 0.3	<1.8	>2.4
DIPA	11.7 ± 1.7	<10.0	>13.4
DMEA	65.7 ± 9.4	<56.3	>75.1

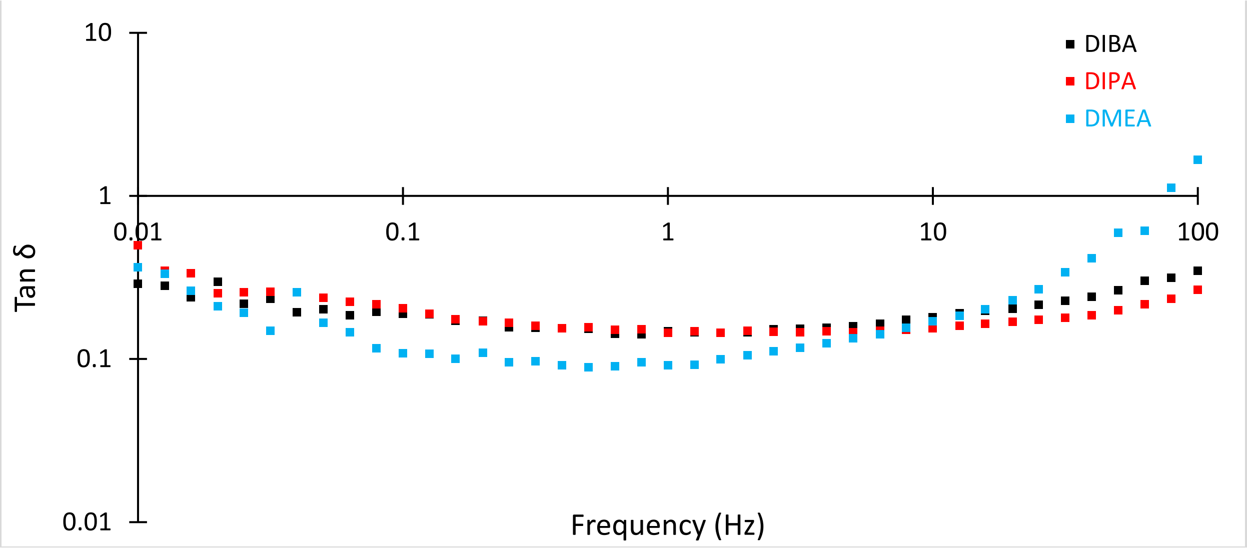

Figure S26. Log-log tan δ versus frequency sweeps (0.01% strain) at 25 ˚C: 3 wt % DIBA-silicone oil gel (black); 3 wt % DIPA-silicone oil gel (red); 3 wt % DMEA-silicone oil gel (blue).

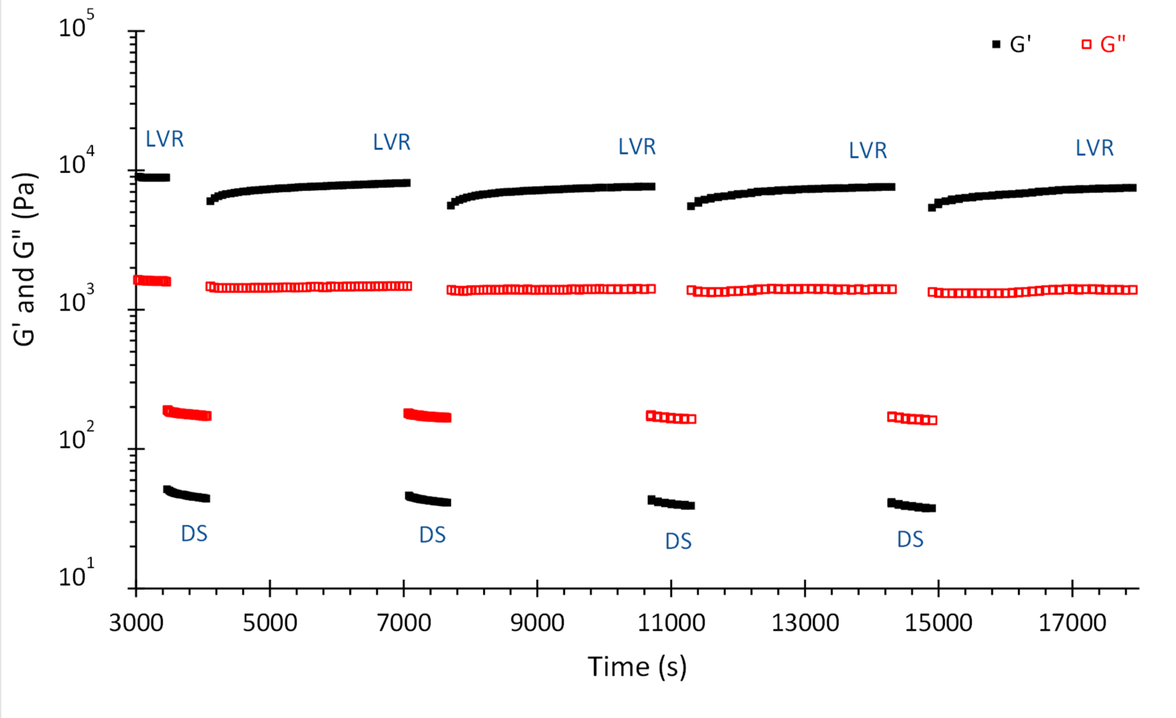

Figure S27. G′ and G″ at 25 ˚C as a function of time for a 3 wt % DIBA in silicone oil gel on alternating application of different strains at room temperature. Linear viscoelastic region (LVR; $\gamma = 0.1$ %, f = 1 Hz) and destructive strain region (DS; $\gamma = 100$ %, f = 1 Hz); repeated four times. Data points were collected every 60 s after the cessation of the destructive strain.

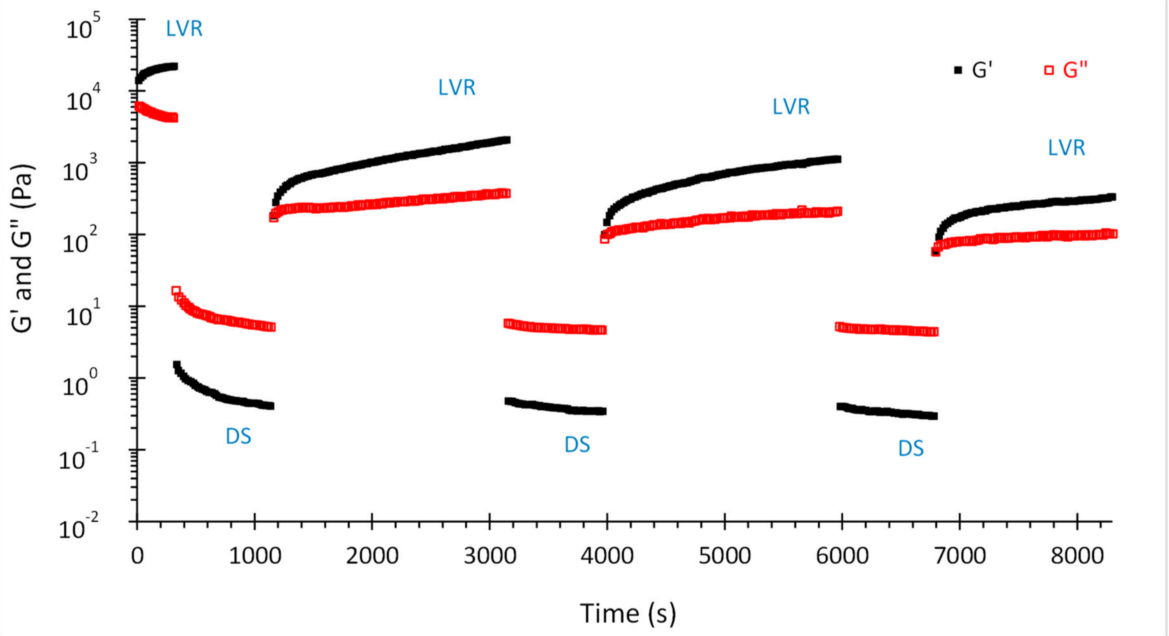

Figure S28. G′ and G″ at 25 ˚C as a function of time for a 3 wt % DIBA in silicone oil gel on alternating application of different strains at room temperature. Linear viscoelastic region (LVR; γ = 0.01 %, f = 1 Hz) and destructive strain region (DS; γ = 700 %, f = 1 Hz); repeated three times. Data points were collected every 20 s after the cessation of the destructive strain.

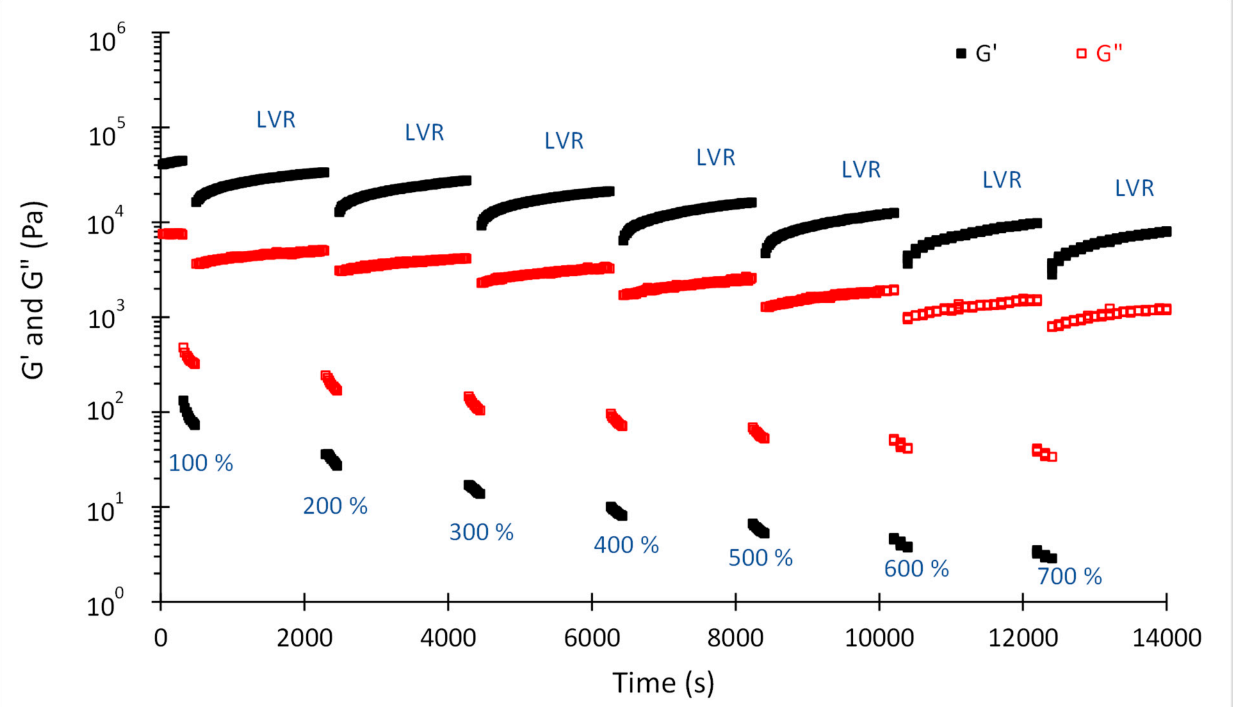

Figure S29. G′ and G″ at 25 ˚C as a function of time for a 3 wt % DIBA in silicone oil gel on alternating application of different strains at room temperature. Linear viscoelastic region (LVR; γ = 0.01 %, f = 1 Hz) and destructive strain region (DS; γ = 100, 200, 300, 400, 500, 600, 700 %, f = 1 Hz); repeated three times. Data points were collected every 20 s after the cessation of the destructive strain.

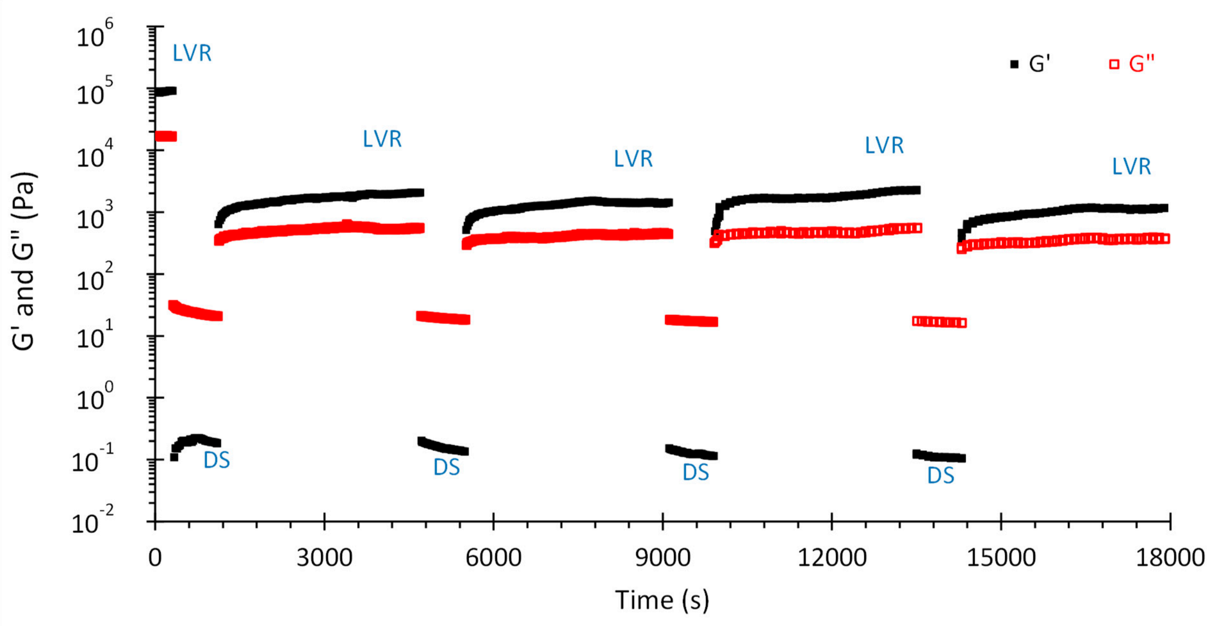

Figure S30. G′ and G″ at 25 ˚C as a function of time for a 3 wt % DIPA in silicone oil gel on application of alternating different strains at room temperature. Linear viscoelastic region (LVR; γ = 0.01 %, f = 1 Hz) and destructive strain region (DS; γ = 600 %, f = 1 Hz); repeated four times. Data points were collected every 20 s after the cessation of the destructive strain.

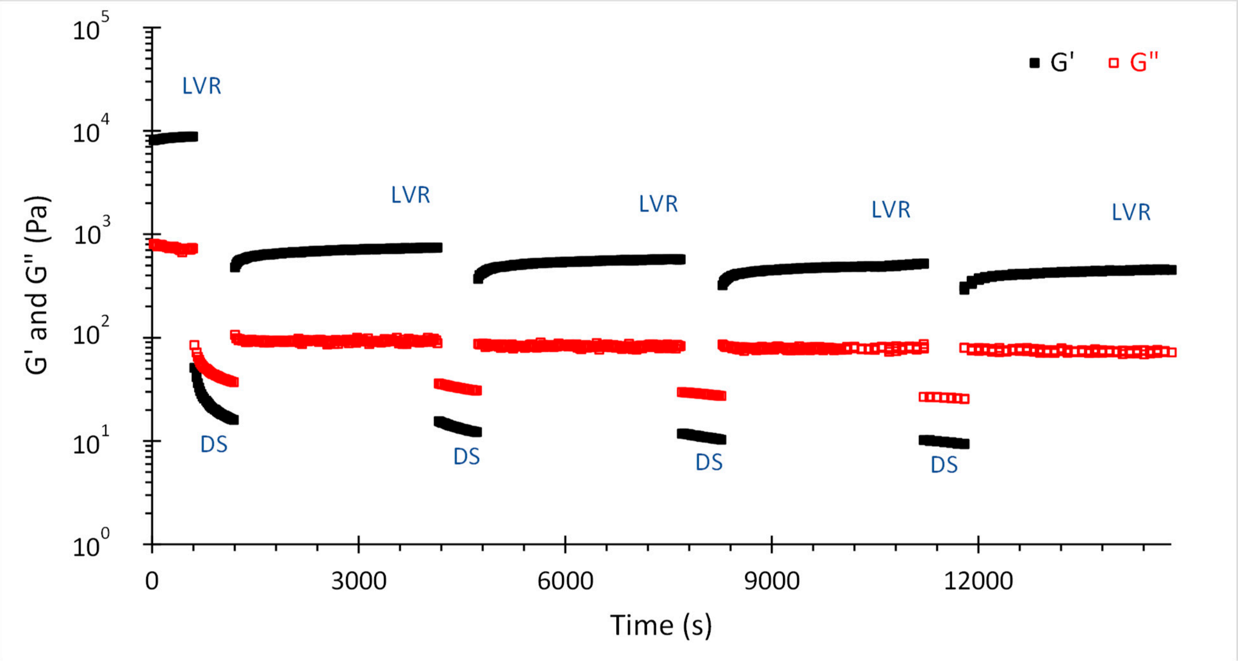

Figure S31. G′ and G″ at 25 ˚C as a function of time for a 3 wt % DMEA in silicone oil gel on alternating application of different strains at room temperature. Linear viscoelastic region (LVR; γ = 0.01 %, f = 1 Hz) and destructive strain region (DS; γ = 100 %, f = 1 Hz); repeated four times. Data points were collected every 20 s after the cessation of the destructive strain.

Table S10. Percentage recovery of viscoelastic properties of 3 wt % gelators in silicone oil (gels) after repeated application of DS and recovery in the LVR. The percentage recovery of the G′ values in each cycle is reported as the G′ value of the preceding cycle as 100 %.

gel (3 wt %)	LVR (strain %)	DS (strain %)	percentage recovery of G′(%)			
			cycle 1	cycle 2	cycle 3	cycle 4
DIBA	0.1	100	92	94	100	98

DIBA	0.01	700	9	54	30	-
DIPA	0.01	600	2	69	159*	51
DMEA	0.01	100	9	77	91	86

*The viscoelastic properties (i.e., the magnitude of G′ was not completely recovered upon cessation of the destructive strain within the time frame of cycle 2; thus, the % recovery of cycle 3 is higher than cycle 2 (which is considered 100 %)).

Table S11. Percentage recovery of viscoelastic properties of 3 wt % DIBA in silicone oil gel after repeated destruction of the gel network on applying variable DS and recovery in the LVR.

DS	Recovery with respect to previous cycle[a] (%)	Recovery with respect to initial G′ value[b] (%)
100	75	75
200	82	62
300	77	48
400	76	36
500	77	28
600	78	22
700	84	18

[a]Percentage recovery of the G′ values in each cycle are reported with reference to the G′ values of the preceding cycle as 100 %.
[b]Percentage recovery of the G′ values in each cycle are reported using the initial G′ value (before applying DS) as 100 %.

Photophysical studies

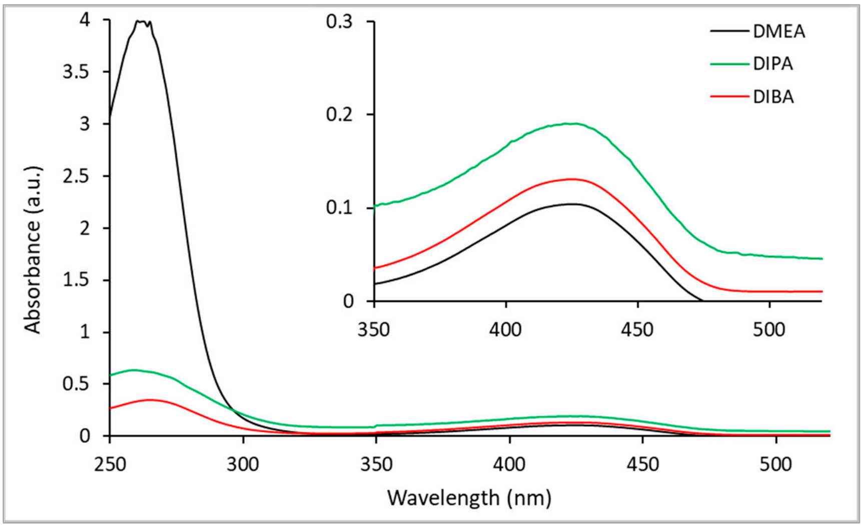

Figure S32. UV-vis absorbance spectra of $6X10^{-3}$ M gelator in acetonitrile: DMEA (black), DIPA (green) and DIBA (red). All measurements were performed at room temperature in 1X1 cm (optical path) quartz cuvettes.

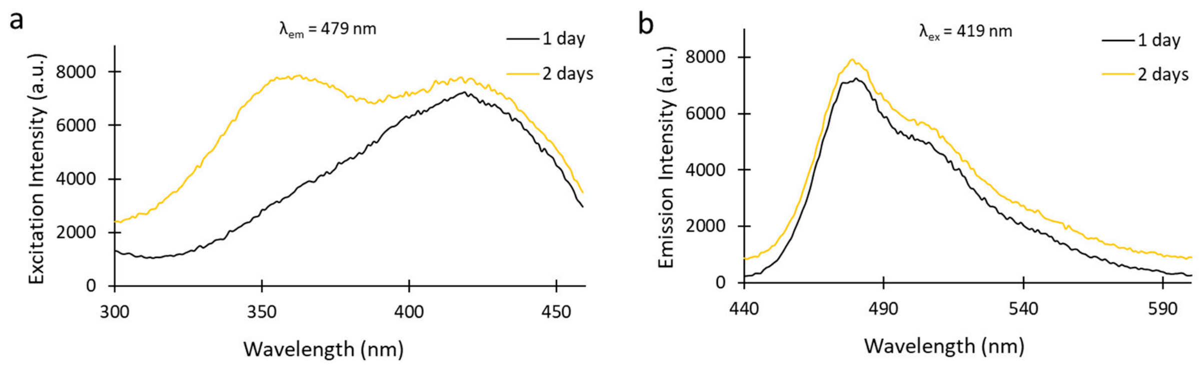

Figure S33. Comparison of photophysical properties of 6X10^{-3} M DMEA in acetonitrile at room temperature in a 1X1 cm (optical path) quartz cuvette recorded within 1 day after the solution was prepared (black, solid line) and 2 days after the solution was prepared (yellow, solid line): a) excitation spectra (λ_{em} = 479 nm); b) emission spectra (λ_{ex} = 419 nm).

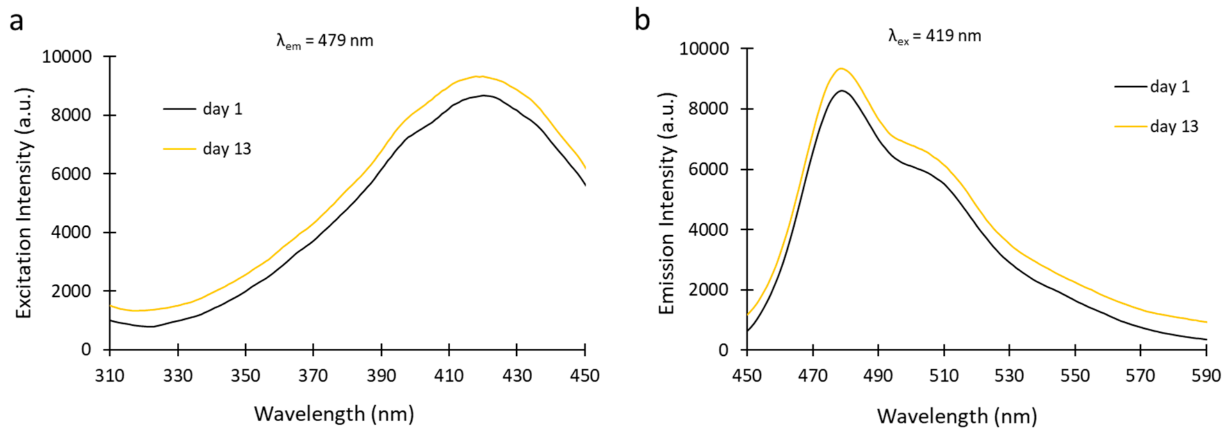

Figure S34. Comparison of photophysical properties of 6X10^{-3} M DIPA in acetonitrile at room temperature in a 1X1 cm (optical path) quartz cuvette recorded within 1 day after the solution was prepared (black, solid line) and 13 days after the solution was prepared (yellow, solid line): a) excitation (λ_{em} = 479 nm); b) emission (λ_{ex} = 419 nm).

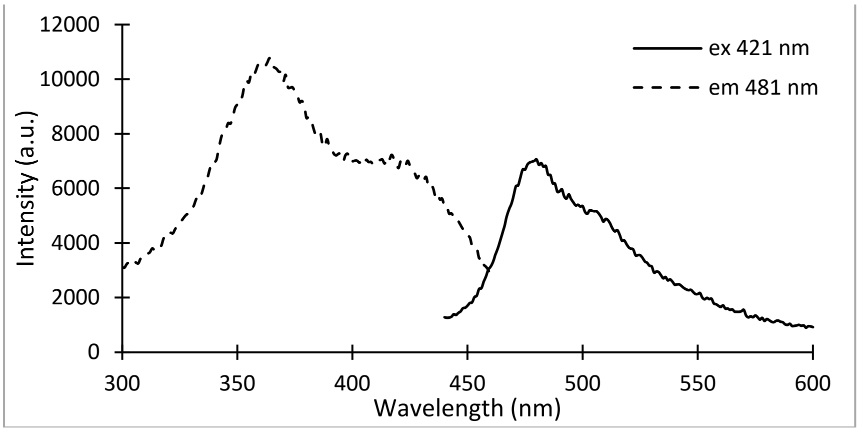

Figure S35. Excitation and emission spectra at room temperature of 6 X 10^{-3} M DMEA in acetonitrile (λ_{em} = 481 nm, dotted line and λ_{ex} = 421 nm, solid line) obtained 4 days after the solution (freeze-pump-thawed) was prepared. Samples in a 5 mm (optical path) flattened pyrex capillary placed at a 45° angle with respect to the incident beam and the detector direction (transmission mode).

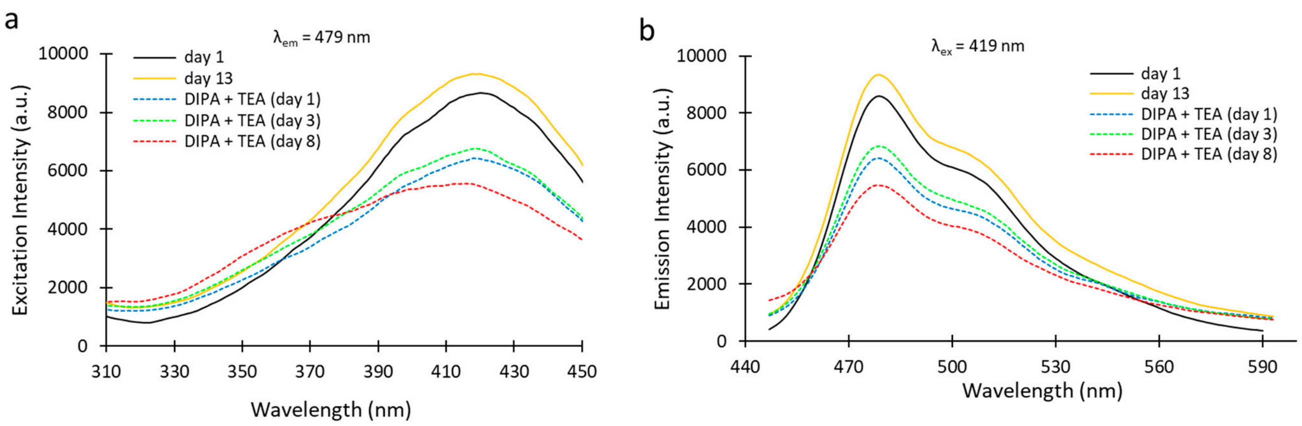

Figure S36. Excitation (**a**) and emission (**b**) spectra of $6X10^{-3}$ M DIPA in acetonitrile at room temperature in a 1X1 cm (optical path) quartz cuvette recorded on the same day as sample preparation (black, solid line), 13 days after preparation (yellow, solid line) and upon addition of triethylamine 13 days after solution preparation. Spectra were also recorded 1 day (blue, dashed line), 3 days (green, dashed line) and 8 days (red, dashed line) after addition of trimethylamine.

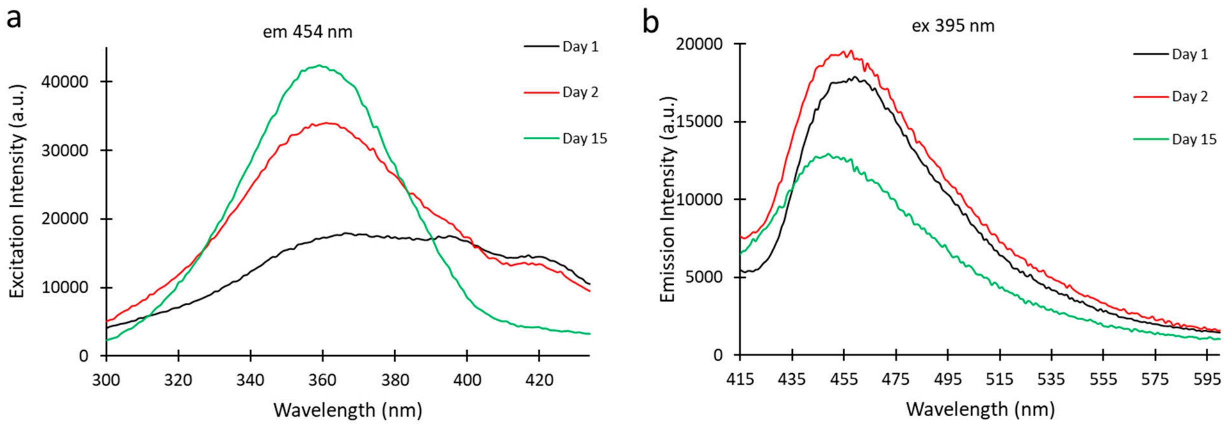

Figure S37. Excitation (**a**) and emission (**b**) spectra of a 3 wt % DMEA in silicone oil gel at room temperature in a 2 mm (optical path) flattened pyrex capillary: recorded during day 1 (black), during day 2 (red) and during day 15 (green) after forming a gel. The capillary was placed at a 45° angle with respect to the incident beam and the detector direction (transmission mode).

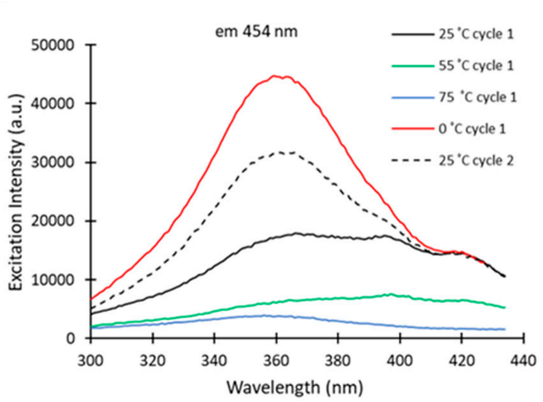

Figure S38. Excitation spectra (λ_{em} = 454 nm) of a 3 wt % DMEA in silicone oil gel in a 2 mm (optical path) flattened pyrex capillary at room temperature (black), at 55 °C (red), at 75 °C (blue) and at 0 °C (green) measured sequentially within one day after forming the gel. The 25 °C measurements were repeated, with cycle 1 represented as a solid line and cycle 2 as a dashed line. The capillary was placed at a 45° angle with respect to the incident beam and the detector direction (transmission mode).

References

1. Zhang, M.; Weiss, R. G. Mechano-Responsive, Thermo-Reversible, Luminescent Organogels Derived from a Long-Chained, Naturally Occurring Fatty Acid. *Chem. Eur. J.* **2016**, *22*, 8262-8272; DOI:10.1002/chem.201600225.
2. Gómez-Zavaglia, A.; Fausto, R. Matrix-Isolation and Solid State Low Temperature FT-IR Study of 2,3-butanedione (diacetyl). *J. Mol. Struct.* **2003**, *661-662*, 195-208; DOI:10.1016/j.molstruc.2003.06.003.
3. Mallia V. A.; George, M.; Blair, D. L.; Weiss, R.G. Robust Organogels from Nitrogen-Containing Derivatives of (R)-12-Hydroxystearic Acid as Gelators: Comparisons with Gels from Stearic Acid Derivatives. *Langmuir* **2009**, *25*, 8615-8625; DOI:10.1021/la8042439.
